# PDIP38/PolDIP2 controls the DNA damage tolerance pathways by increasing the relative usage of translesion DNA synthesis over template switching

**DOI:** 10.1371/journal.pone.0213383

**Published:** 2019-03-06

**Authors:** Masataka Tsuda, Saki Ogawa, Masato Ooka, Kaori Kobayashi, Kouji Hirota, Mitsuo Wakasugi, Tsukasa Matsunaga, Tetsushi Sakuma, Takashi Yamamoto, Shunsuke Chikuma, Hiroyuki Sasanuma, Michelle Debatisse, Aidan J. Doherty, Robert P. Fuchs, Shunichi Takeda

**Affiliations:** 1 Department of Radiation Genetics, Graduate School of Medicine, Kyoto University, Kyoto, Japan; 2 Department of Mathematical and Life Sciences, Graduate School of Science, Hiroshima University, Hiroshima, Japan; 3 Department of Chemistry, Graduate School of Science, Tokyo Metropolitan University, Hachioji-shi, Tokyo, Japan; 4 Faculty of Pharmacy, Institute of Medical, Pharmaceutical and Health Sciences, Kanazawa University, Kanazawa, Japan; 5 Department of Microbiology and Immunology, Keio University School of Medicine, Tokyo, Japan; 6 Institut Curie UMR 3244, Universite Pierre et Marie Curie (Paris 06), CNRS Paris, France; 7 Genome Damage and Stability Centre, School of Life Sciences, University of Sussex, Brighton, United Kingdom; 8 DNA Damage Tolerance CNRS, UMR7258, Marseille, France; 9 Institut Paoli-Calmettes, Marseille, France; 10 Aix-Marseille University, UM 105, Marseille, France; 11 Inserm, U1068, CRCM, Marseille, France; University of Minnesota Twin Cities, UNITED STATES

## Abstract

Replicative DNA polymerases are frequently stalled at damaged template strands. Stalled replication forks are restored by the DNA damage tolerance (DDT) pathways, error-prone translesion DNA synthesis (TLS) to cope with excessive DNA damage, and error-free template switching (TS) by homologous DNA recombination. PDIP38 (Pol-delta interacting protein of 38 kDa), also called Pol δ-interacting protein 2 (PolDIP2), physically associates with TLS DNA polymerases, polymerase η (Polη), Polλ, and PrimPol, and activates them *in vitro*. It remains unclear whether PDIP38 promotes TLS *in vivo*, since no method allows for measuring individual TLS events in mammalian cells. We disrupted the *PDIP38* gene, generating *PDIP38*^*-/*-^ cells from the chicken DT40 and human TK6 B cell lines. These *PDIP38*^*-/*-^ cells did not show a significant sensitivity to either UV or H_2_O_2_, a phenotype not seen in any TLS-polymerase-deficient DT40 or TK6 mutants. DT40 provides a unique opportunity of examining individual TLS and TS events by the nucleotide sequence analysis of the immunoglobulin variable (Ig V) gene as the cells continuously diversify Ig V by TLS (non-templated Ig V hypermutation) and TS (Ig gene conversion) during *in vitro* culture. *PDIP38*^*-/*-^ cells showed a shift in Ig V diversification from TLS to TS. We measured the relative usage of TLS and TS in TK6 cells at a chemically synthesized UV damage (CPD) integrated into genomic DNA. The loss of PDIP38 also caused an increase in the relative usage of TS. The number of UV-induced sister chromatid exchanges, TS events associated with crossover, was increased a few times in *PDIP38*^*-/*-^ human and chicken cells. Collectively, the loss of PDIP38 consistently causes a shift in DDT from TLS to TS without enhancing cellular sensitivity to DNA damage. We propose that PDIP38 controls the relative usage of TLS and TS increasing usage of TLS without changing the overall capability of DDT.

## Introduction

DNA replication is a fragile system and is frequently stalled at damaged template strands. Stalled replication forks are released by the two major DDT pathways, TLS and TS. Error-free TS is expected to play the dominant role in the DDT during the physiological cell cycle, while error-prone TLS needs to be strongly activated when excess amounts of DNA lesions are induced upon exposure to environmental genotoxic agents such as UV [1, 2]. Homologous recombination (HR) facilitates transient switching of replication primers from the damaged template strand to the newly synthesized sister chromatid via a mechanism that remains very poorly understood even in *Saccharomyces cerevisiae* (*S*. *cerevisiae*) [3–5]. TLS is carried out by a number of specialized DNA polymerases, called TLS polymerases, such as Polη, Polκ, Polλ, Polν, Polθ, Polζ, PrimPol, and Rev1 [6, 7]. Polη plays the dominant role in TLS past UV lesions, and bypass them with high accuracy [8]. TLS is controlled by a number of mechanisms in higher eukaryotes. First, Proliferating Cell Nuclear Antigen (PCNA), a molecular sliding clamp for replicative DNA polymerase, plays the central role in a molecular switch from replicative polymerases to TLS polymerases [9]. In response to replication blockage, the mono-ubiquitination of PCNA by Rad18 ubiquitylation enzyme facilitates the recruitment of TLS polymerases to stalled replication forks [10]. Second, Rev1 facilitates TLS by associating with several TLS polymerases such as Polη, Polκ, Polι, Polζ [11].

PDIP38, also called PolDIP2, was initially shown to interact with POLD2 (p50), a subunit of both Polδ and Polζ [12–14]. PDIP38 also interacts with PCNA [13], suggesting that PDIP38 might be an integral component of the replication machinery. Yeast two hybrid experiment demonstrated that PDIP38 directly interacts with Polη, Polζ, and Rev1 [15]. Purified PDIP38 stimulates TLS by Polλ and PrimPol *in vitro* [16, 17]. These data suggest that PDIP38 may promote TLS by stimulating the activity of these TLS polymerases. However, the role played by PDIP38 in TLS has not yet been verified *in vivo* due to technical difficulty of measuring individual TLS events in mammalian cells.

Two methods have been established for measuring the usage of TLS and TS following replication blockage at defined lesions. First, like primary chicken B lymphocytes, the DT40 B cell line diversifies Ig V gene by both TLS and TS during *in vitro* culture, and provides a unique opportunity of measuring the number of TLS and TS events at the Ig V gene [18, 19]. The avian Ig V diversification is triggered by activation-induced deaminase (AID) mediated conversion of dC to dU at the Ig V_λ_ segment followed by formation of the abasic (AP) site (S1A Fig) [20, 21], the most common spontaneously-arising lesion in the chromosomal DNA [22]. The abasic site blocks replication fork progression, which blockage is released by TLS past abasic sites and by TS. The TS at Ig V is mediated by intragenic HR between the Ig V_λ_ segment and a set of homologous upstream pseudo-V_λ_ segments (S1B Fig)[23]. TLS and TS lead to non-templated single base substitutions at dG/dC pairs (Ig V hypermutation) and templated mutagenesis (Ig gene conversion), respectively [19, 20, 24, 25]. The chicken DT40 B cell line continuously undergoes Ig V diversification during *in vitro* passage, and thus provides a unique opportunity of phenotypically examining individual TLS and TS events on a nucleotide sequence level. The second method employs the random integration of UV damage (CPD) into the genome of cells using the ‘piggyBlock’ transposon-based vector assay (S2 Fig)[26, 27]. This method allows for accurately measuring the relative usage of TLS and TS for bypassing the CPD site on the genomic DNA.

We here examined the capability of DDT pathways in *PDIP38*^*-/*-^ cells derived from the human TK6 and chicken DT40 B cell lines. Although neither human nor chicken *PDIP38*^*-/*-^ cells show increased sensitivity to H_2_O_2_ or UV, these cells displayed a decrease in the frequency of TLS associated with an increase in the frequency of TS. The loss of PDIP38 increased UV sensitivity of TK6 cells only in the absence of Polη, the major TLS polymerase in UV tolerance. Unlike *PDIP38*^*-/-*^ cells, *POLλ*^*-/-*^, and *PRIMPOL*^*-/-*^ cells show increased sensitivity to H_2_O_2_ and UV, respectively [28, 29]. These data indicate that PDIP38 can increase the usage of TLS independently of Polλ, Polη and PrimPol. We propose that PDIP38 controls DDT by shifting the relative usage of DDT pathway from TS to TLS without affecting the overall capability of DDT pathways.

## Materials and methods

### Cell culture

The DT40 cell line was derived from chicken B lymphoma [30] and was cultured in RPMI 1640 medium (Nacalai Tesque, Kyoto, Japan) supplemented with 10% heat-inactivated FBS (fetal bovine serum), 1% chicken-serum, 50 μM mercaptoethanol (Nacalai Tesque), L-glutamine (Nacalai Tesque), 50 U/ml penicillin, and 50 μg/ml streptomycin (Nacalai Tesque). The cell line was maintained at 39.5°C in a humidified atmosphere and 5% CO_2_. TK6 cell line is a human B lymphoblastoid line [31] and cultured in RPMI 1640 medium (Nacalai Tesque, Kyoto, Japan) supplemented with 5% heat-inactivated horse-serum, L-glutamine (Nacalai Tesque), 0.2 mg/ml Sodium pyruvate (Sigma-Aldrich), 50 U/ml penicillin, and 50 μg/ml streptomycin (Nacalai Tesque). The TK6 cells were maintained at 37°C in a humidified atmosphere and 5% CO_2_. The list of the mutant clones we analyzed in this manuscript is shown in Table 1.

**Table 1:** List of gene-disrupted cells.

Genotype	Name of Cell line and Species	Marker Genes	Reference
*BRCA2*^*-/-*^	Chicken DT40	*bsr*^*R*^, *puro*^*R*^	[32]
*PDIP38*^*-/-*^	Chicken DT40	*bsr*^*R*^, *puro*^*R*^	This study
*PDIP38*^*-/-*^*/POLη*^*-/-*^	Chicken DT40	*bsr*^*R*^, *puro*^*R*^, *neo*^*R*^, *ecogpt*	This study
*PIF1*^*-/-*^	Chicken DT40	*bsr*^*R*^, *neo*^*R*^	[33]
*POLη*^*-/-*^	Chicken DT40	*bsr*^*R*^, *puro*^*R*^	[25]
*POLλ*^*-/-*^	Chicken DT40	*bsr*^*R*^, *puro*^*R*^	[29]
*PRIMPOL*^*-/-*^	Chicken DT40	*bsr*^*R*^, *puro*^*R*^	[34]
*PDIP38*^*-/-*^	Human TK6	*neo*^*R*^, *hygro*^*R*^	This study
*POLη*^*-/-*^	Human TK6	*puro*^*R*^, *neo*^*R*^	[35]
*POLλ*^*-/-*^	Human TK6	*puro*^*R*^, *neo*^*R*^	This study
*PRIMPOL*^*-/-*^	Human TK6	*hygro*^*R*^	This study
*RAD54*^*-/-*^	Human TK6	*puro*^*R*^, *neo*^*R*^	[36]
*XPA*^*-/-*^	Human TK6	*hygro*^*R*^, *his*^*R*^	[35]
*PDIP38*^*-/-*^*/POLη*^*-/-*^	Human TK6	*puro*^*R*^, *neo*^*R*^, *hygro*^*R*^, *his*^*R*^	This study
*XPA*^*-/-*^*/PDIP38*^*-/-*^	Human TK6	*neo*^*R*^, *hygro*^*R*^	This study
*XPA*^*-/-*^*/POLη*^*-/-*^	Human TK6	*puro*^*R*^, *neo*^*R*^, *hygro*^*R*^, *his*^*R*^	This study
*XPA*^*-/-*^*/PDIP38*^*-/-*^*/POLη*^*-/-*^	Human TK6	*neo*^*R*^, *hygro*^*R*^	This study

### Generation of *PDIP38*^*-/-*^ mutant DT40 cells

We created the *PDIP38* gene disruption constructs, *PDIP38-bsr*^*R*^, *PDIP38-puro*^*R*^, *PDIP38-neo*^*R*^, and *PDIP38-ecogpt*, by combining left and right arms generated from genomic PCR products with the *bsr*^*R*^, *puro*^*R*^, *neo*^*R*^, and *ecogpt* selection marker genes (S3A Fig). We amplified genomic DNA sequences using the following primers: 5’-GGCACCTCTCGTCTCGGTGAGGC -3’ and 5’-CTATTAGCACCTGATAGTAAGTATG -3’ for the left arm, and 5’-AGACTATGTAAGCCATGAAGATATCC -3’ and 5’-GGATGCCTGCAGGGAGACGTGACTGCTGTAC -3’ for the right arm. Amplified PCR products (2.1 kb left and 2.5 kb right arms) were cloned separately into the pCR-Blunt II-TOPO vector (Invitrogen, Carlsbad, CA). We isolated the 2.5-kb *Kpn*I fragment, and cloned into the *Kpn*I site of pCR-Blunt II-TOPO vector containing the 2.1 kb left arm sequence. The *BamH*I site of the resulting plasmid was used to insert selection marker genes flanked by *loxP* sequences to generate the *PDIP38-puro*^*R*^, *PDIP38-bsr*^*R*^, *PDIP38-neo*^*R*^, and *PDIP38-ecogpt* gene-disruption constructs. For the preparation of a probe for Southern blot analysis of gene-disrupted clones, a 0.5 kb genomic DNA fragments was amplified by the following primers: 5’-GGTAGACTCAGTTCCTCCAC-3’ and 5’-GAGAAAAGGGTGGGATTCGG-3’. The disruption constructs were linearized with *Not*I prior to transfection. *Wild-type* cells were sequentially transfected with the *PDIP38-puro*^*R*^ and *PDIP38-bsr*^*R*^ targeting constructs to obtain *PDIP38*^*-/-*^ DT40 cells. The loss of *PDIP38* transcript was confirmed by RT-PCR using primers: 5’- GATACACTTTGTGCATGGCAGGAAAAG -3’ and 5’- CTGAAATGCTGGCTGTTCTTTAGATAACAC -3’. *β-actin* transcripts were analyzed as a positive control for the RT-PCR analysis using primers: 5’- GATGATGATGATATTGCTGCGCTCGTTGTTGAC -3’ and 5’- GATTCATCGTACTCCTGCTTGCTGATCCAC -3’.

### Generation of *PDIP38*^*-/-*^ mutant TK6 cells

We created the *PDIP38* gene disruption constructs, *PDIP38-neo*^*R*^ and *PDIP38-hygro*^*R*^, by combining left and right arms with *neo*^*R*^ and *hygro*^*R*^ selection marker genes. To generate these arms, we amplified genomic DNA sequences using the following primers: 5’-TAGGATATTGTAGGTAAGGA-3’ and 5’-TGGGAGAAGGAGGCCAAGAT-3’ for the left arm, and 5’- TAAGCAAGATGGCTGGGCTT-3’ and 5’- ACAGGAGGTTGAAAAGGGTT-3’ for the right arm. The DT-A-pA/loxP/PGK-*neo*^*R*^-pA/loxP vector was provided by Laboratory for Animal Resources and Genetic Engineering, Center for Developmental Biology, Institute of Physical and Chemical Research, Kobe, Japan. The left and right arms were inserted into *Apa*I and *Afl*II sites of DT-A-pA/loxP/PGK-*neo*^*R*^-pA/loxP [36], respectively, to create the *PDIP38-neo*^*R*^ using GENEART Seamless Cloning (Life Technologies, Palo Alto, CA). *PDIP38-hygro*^*R*^ was generated using DT-A-pA/loxP/PGK-*hygro*^*R*^-pA/loxP [36] with the same method as for generation of *PDIP38-neo*^*R*^. We increased the gene targeting efficiency by using transcription activator-like effector nuclease (TALEN) [37]. The TALEN plasmids were constructed using the Platinum Gate TALEN Kit (Addgene, Cambridge, MA). Target genomic sequences of TALENs in the human PDIP38 gene are shown in S4 Fig (*PDIP38*-TALEN-L and *PDIP38*-TALEN-R). We simultaneously transfected 2 μg each of targeting vectors (*PDIP38-neo*^*R*^ and *PDIP38-hygro*^*R*^) and 6 μg each of TALEN expressing vectors (*PDIP38-TALEN-L* and *PDIP38*-*TALEN-R*) into TK6 cells using NEON Transfection System (Life Technologies). At 48 h, the cells were plated in 96-well plates, and then subjected to the selection with both hygromycin (0.6 mg/ml) and neomycin (1 mg/ml). The drug-resistant cell colonies were picked up on days 7–10 after transfection. We prepared a probe for Southern blot analysis of gene-disrupted clones, a 0.5 kb genomic DNA fragment, by amplifying genomic DNA using the following primers: 5’-ATCGATCTGGACACAAGGAGGGGACCCCGG-3’ and 5’-CGGGCTGTACAGGCTGCCATGTCCCGCCCG-3’. The loss of *PDIP38* transcript was confirmed by RT-PCR using primers 5’- CTGATTGATGCTCGTGACTGCCCACATATA -3’ and 5’- TATGTTCTCAGTTGTTTCCCGATGAACATC -3’. *GAPDH* transcripts were analyzed as a positive control for the RT-PCR analysis using primers 5’- TGGCCAAGGTCATCCATGACAACTT-3’ and 5’- GCGCCAGTAGAGGCAGGGATGATGT -3’. We transfected the targeting vectors (*PDIP38-hygro*^*R*^ and *PDIP38-his*^*R*^) together with TALEN expressing vectors (*PDIP38-TALEN-L* and *PDIP38*-*TALEN-R*) into *POLη*^*-/-*^ TK6 cells to obtain *PDIP38*^*-/-*^*/POLη*^*-/-*^ TK6 cells.

### Generation of *POLλ*^*-/-*^ mutant TK6 cells

We created the *POLλ* gene disruption constructs, *POLλ-neo*^*R*^ and *POLλ-puro*^*R*^, by combining left and right arms with *neo*^*R*^ and *puro*^*R*^ selection marker genes (S5 Fig). To obtain the arm fragments, we amplified genomic DNA using the primers: 5’-AATCACAACCTCCATATCAC-3’ and 5’-GCTTCCCAATCCCAGGGATA-3’ for the left arm, and 5’-CCTACTTCAGTTTTGCTGTG-3’ and 5’-TAACCCAATCCTAACACCAA-3’ for the right arm. The left and right arms were inserted into *Apa*I and *Afl*II site of DT-A-pA/loxP/PGK-*neo*^*R*^-pA/loxP, respectively, to create *POLλ-neo*^*R*^ using GENEART Seamless Cloning. *POLλ-puro*^*R*^ was generated using DT-A-pA/loxP/PGK-*puro*^*R*^-pA/loxP [36] with the same method as for generation of *POLλ-neo*^*R*^. We increased gene targeting efficiency by using clustered regularly interspaced short palindromic repeat (CRISPR) [38]. Guide RNA sequences, 5’-GAGCGGGCATTTGCGGAAGC-3’, was inserted into the pX330 vector. We transfected 2 μg each of targeting vectors (*POLλ-neo*^*R*^ and *POLλ-puro*^*R*^) and 6 μg of the guide sequence-containing pX330 vector into TK6 cells using NEON Transfection System. At 48 h, the cells were plated in 96-well plates and subjected to the drug selection with both puromycin (0.5 μg/ml) and neomycin (1 mg/ml). The drug-resistant cell colonies were picked on days 7–10 after transfection. The loss of *POLλ* transcript was confirmed by RT-PCR using primers 5’-ATGGCTGAGAAAATCATAGAGATCCTGGAG-3’ and 5’-AGTACTTCTGTTGCTGACCATTCTCCTCTT-3’.

### Generation of *PRIMPOL*^*-/-*^ mutant TK6 cells

We created the *PRIMPOL* gene disruption construct, *PRIMPOL-hygro*^*R*^, by combining left and right arms with the *hygro*^*R*^ gene (S6 Fig). To generate the arm fragments, we amplified genomic DNA using the primers: 5’-CCTCGAGATTTGCCAATGAGTTTGTGTTGCTGCAAAG -3’ and 5’-GGCTAGCTGCTTCAGTTTTGCTTCCCATTT-3’ for the left arm, 5’- GGCGGCCGCCTGGAGACTATTTCATCGACAAGCTCAAGC -3’ and 5’- CCTTAAGTTGTCCATCTCCTACTTTGCATTCCAAAGC -3’ for the right arm. Note that the underlined sequences denote the restriction enzyme sites (*Xho*I, *Nhe*I, *Not*I, and *Afl*ll). Amplified PCR products (1.5 kb left and 2.1 kb right arms) were separately cloned into the pCR-Blunt II-TOPO vector. The 1.5 kb *Xho*I-*Nhe*I fragment and the 2.1 kb were cloned into *Xho*I-*Nhe*I and *Not*I-*Afl*II sites, respectively, of DT-A-pA/loxP/PGK-*hygro*^*R*^-pA/loxP using GENEART Seamless Cloning kit to create *PRIMPOL-hygro*^*R*^. We increased gene targeting efficiency by using TALEN [37]. Target sequences of TALENs are shown in S6 Fig. We transfected 2 μg each of targeting vector (*PRIMPOL-hygro*^*R*^) and 6 μg each of TALEN expressing vectors (*PRIMPOL-TALEN-L* and *PRIMPOL*-*TALEN-R*) into TK6 cells using NEON Transfection System. At 48 h, the cells were plated in 96-well plates, and then subjected to the selection with hygromycin (0.6 mg/ml). The drug-resistant cell colonies were picked on days 7–10 after transfection. The loss of *PRIMPOL* transcript was confirmed by RT-PCR using primers 5’- ATGAATAGAAAATGGGAAGCAAAACTG -3’ and 5’- TTACTCTTGTAATACTTCTATAATTAGTT -3’. *β-actin* transcripts were analyzed as a positive control for the RT-PCR analysis using primers 5’- GATGGTGGGCATGGGTCAGAAGGATTCC -3’ and 5’- GTCCAGGGCGAGGTAGCACAGCTTCTC -3’.

### cDNA synthesis

Total RNA was extracted, and first strand DNA was prepared by Superscript First-Strand Synthesis System (Invitrogen, CA, USA).

### Measurement of DNA damage sensitivity in DT40 cells

To determine sensitivity to H_2_O_2_ for DT40 cells, 1 × 10^6^ cells were treated for 1 h at 39.5° C in 1ml of complete medium containing H_2_O_2_ and washed with media for removing H_2_O_2_. UV-irradiation was done by suspending 1×10^4^ cells in 30 μl of 1% FBS containing PBS, spreading 30 μl cell suspensions on individual wells of 6-well plates, and exposing cells to UV [25]. After exposure to H_2_O_2_ or UV, 1×10^4^ cells were suspended in 1 ml of complete medium and incubated for 48 h. We exposed 1×10^4^ cells to olaparib, aphidicolin, and cisplatin included in 1 ml complete medium for 48 h. To measure cellularity, we transferred 100 μl of cell suspension to 96-well plates and measured the amount of ATP in cellular lysates using the CellTiter-Glo (Promega) kit. Luminescence was measured by Multilabel Plate Reader ARVO X5 (PerkinElmer Inc, Waltham, MA)[39].

### Measurement of DNA damage sensitivity in TK6 cells

To determine sensitivity to H_2_O_2_ for TK6 cells, 1 × 10^6^ cells were treated for 1 h at 37° C in 1 ml of complete medium containing H_2_O_2_ and washed with media for removing H_2_O_2_. To determine sensitivity to UV for TK6 cells, 1×10^3^ cells were suspended in 30 μl of 1% FBS in PBS, spread cell suspensions on individual wells of 6-well plates, and irradiated with various doses of UV. We plated serially diluted TK6 cells in triplicate onto 6-well plates with 5 ml/well of 1.5% (w/v) methylcellulose (Sigma-Aldrich, St. Louis, MO) containing Dulbecco’s modified Eagles’s medium/F-12 (Invitrogen), 10% horse serum (Life Technologies). To measure olaparib and aphidicolin sensitivity, we inoculated step-wise diluted cells into the above methylcellulose-containing media containing various concentrations of olaparib and aphidicolin. The number of colonies was counted at day 10 to 14 [39].

### *In vivo* nucleotide excision repair assay

Cultured TK6 cells were washed with PBS (-) and resuspended in PBS (-) containing 1% FBS. The cells (5 ×10^6^) were transferred to a 60-mm dish and irradiated with 20 J/m^2^ of UV-C from germicidal lamps. After various repair incubation, genomic DNAs were purified using DNeasy kit (Qiagen). The amount of 6-4PP was determined by an enzyme-linked immunosorbent assay (ELISA) with 64M-5 monoclonal antibody [40].

### Chromosome aberration analysis

Preparation of chromosome samples and karyotype analysis of DT40 and TK6 cells were performed as described previously [41]. For the enrichment of mitotic cells, 0.1 μg/ml Colcemid was added to the last 3 h of incubation.

### Analysis of chromosome fragile site

TK6 cells were cultured for 48 h with 100 nM aphidicolin and incubated with 0.1 μg/ml Colcemid for 3 h before collection. The cells were swollen in hypotonic solution (75 mM KCl) and fixed with ethanol-acetic acid (3:1). Fluorescence in situ hybridization (FISH) was carried out as previously described [42]. FRA3B was probed with BAC clones RP11-170K19 and RP11-495E23. Immunodetection was performed by alternating incubations with the following antibodies: streptavidin-Cy3 (1:200) (Invitrogen), biotinylated rabbit anti-streptavidin (1:266)(Rockland, Limerick, PA) and mouse anti-digoxygenin FITC (1:50)(Interchim, Montluçon, France) and goat anti-mouse Alexa 488 (1:200, Invitrogen) The cells were mounted with Vectashield mounting medium containing DAPI (Vector Laboratories, Burlingame, CA).

### Measurement of rate of sister chromatid exchange

Measurement of sister chromatid exchange (SCE) levels was performed as described previously with some modifications [43]. DT40 and TK6 cells (2×10^6^) were cultured for two cycle periods with medium containing 10 μM BrdU and pulsed with 0.1 μg/ml colcemid for two hrs. The cells were harvested and treated with 75 mM KCl for 20 min at room temperature and then fixed with methanol-acetic acid (3:1) for 30 min. The cell suspension was dropped onto glass slides and air-dried. The cells on the slides were incubated with 10 μg/ml Hoechst 33258 in phosphate buffer (pH 6.8) for 20 min and rinsed with MacIlvaine solution (164 mM Na_2_HPO4, 16 mM citric acid, pH 7.0). The cells were exposed to a black light (λ = 352 nm) for 30 min and incubated in 2×SSC (0.3 M NaCl, 0.03 M sodium citrate) at 58°C for 60 min and then stained with 3% Giemsa solution for 25 min. To measure UV-induced SCE, cells were suspended in PBS containing 1% FCS, inoculated in 6-well plates, and irradiated with UV at 0.25 J/m^2^ for DT40 cells or 5 J/m^2^ for TK6 cells. The irradiated cells were cultured for two cycle periods (18 h for DT40 cells or 28 h for TK6 cells) with medium containing 10 μM BrdU and pulsed with 0.1 μg/ml colcemid for the last two hrs.

### Analysis of rate of surface IgM gain analysis

The generation frequency of surface IgM gain revertants was monitored by flow cytometric analysis of cells that had been expanded for three weeks after subcloning and stained with fluorescein isothiocyanate-conjugated goat anti-chicken IgM (BethylLaboratories, Inc.) [44]. 30 subclones were analyzed in each genotype.

### Analysis of Ig V nontemplated point mutations and gene conversion at the VJ_λ_ segment

DNA was extracted from five clones from each genotype at two weeks after AID-expressing virus infection. The PCR-amplified fragments of the V_λ_ segments were cloned into plasmid and subjected to base-sequence analysis. The rearranged VJ_λ_ was amplified using the CVL6 (5’-CAGGAGCTCGCGGGGCCGTCACTGATTGCCG-3’) and CVLR3 (5’-GCGCAAGCTTCCCCAGCCTGCCGCCAAGTCCAAG-3’) primers as previously described [45]. After purification with gel extraction kit (QIAquick; QIAGEN, Venlo, Netherlands), The *EcoR*I-*BamH*I-PCR fragments were cloned into the pBluescriptII and sequenced with the M13 reverse primer and a sequence service (Beckman Coulter, Pasadena, CA). Nucleotide sequence alignment, using GENETYX-MAC (Software Development, Tokyo, Japan), allowed the identification of changes from the parental sequences in each clone. Differentiation between nontemplated nucleotides substitutions and gene conversion was carried out as previously described [45]. The rate of nontemplated point mutation was calculated based on mutation frequency and term of culture (two weeks).

### PiggyBlock assay

A 30-nucleotide oligonucleotide, 5’-CTCGTCAGCATC(TT)CATCATACAGTCAGTG-3’ carrying CPD on (TT), and a 29-nucleotide oligonucleotide, 5’-TCGAGCGACACACTCGCTGACTAGTGGAT-3’, was annealed with complementary 59-nucleotide oligonucleotide, 5’-AATTCACTGACTGTATGATGGCGATGCTGACGAGATCCACTAGTCA(TT)GAGTGTGTCGC-3’. The resultant duplex fragment carrying a two CPD lesion was ligated with the piggyBlock-*Sal*I plasmid [27] digested with *Mfe*I/*Sal*I, and ligated plasmid was gel purified (Qiagen), as previously described [26]. Ten ng of the ligated plasmid together with 1 μg of transposase expression vector was transfected into TK6 cells using the NEON transfection system with settings, 1350 V, 10 msec, and three pulses. Transfected cells were subjected to limiting dilution immediately after transfection. Puromycin was added at 48 h after transfection. Genomic DNAs from individual puromycin resistant clones were purified, and were PCR amplified using primers (5’-ACTGATTTTGAACTATAACGACCGCGTGAG-3’) and (5’-ACTAGTGAGACGTGCTACTTCCATTTGTCA-3’) to examine DNA sequences at the CPD lesion. If a single puromycin resistant clone contained two different sequences, we counted as two independent DNA synthesis events. We analyzed them following the method described previously [26].

## Results

### Disruption of *PDIP38* gene in chicken DT40 and human TK6 B lymphocyte lines

We disrupted the *PDIP38* gene in DT40 and TK6 cell lines (S3 and S4 Figs). The resulting *PDIP38*^*-/*-^ clones analyzed in this study are summarized in Table 1. DT40 cells are deficient in p53, while TK6 cells retain functional p53 [31, 46]. The S-phase cells account for 70% and 50% of the whole cell cycle time of DT40 and TK6 cells, respectively, which high percentages of S-phase cells allow for sensitive detection of defects in DDT pathways functioning during DNA replication. TK6 has been widely used for evaluating the genotoxicity of industrial chemical compounds following the OECD guideline due to the phenotypic stability and tractability of the cells [47]. The *PDIP38*^*-/*-^ clones derived from DT40 and TK6 proliferated with normal kinetics (S3D and S4D Figs). For the phenotypic comparison with *PDIP38*^*-/*-^ TK6 cells, we created *POLλ*^*-/-*^ and *PRIMPOL*^*-/-*^ TK6 cells (S5 and S6 Figs). To explore a functional relationship between Polη and PDIP38, we disrupted the *PDIP38* gene in *POLη*^*-/-*^ DT40 and TK6 cells. The resulting *PDIP38*^*-/-*^*/POLη*^*-/-*^ DT40 and TK6 cells proliferated with normal kinetics (S3D and S4D Figs).

### DNA damage sensitivity profile differs between *PDIP38*^*-/-*^ cells and those deficient in Polη, Polλ and PrimPol

**Fig 1:**
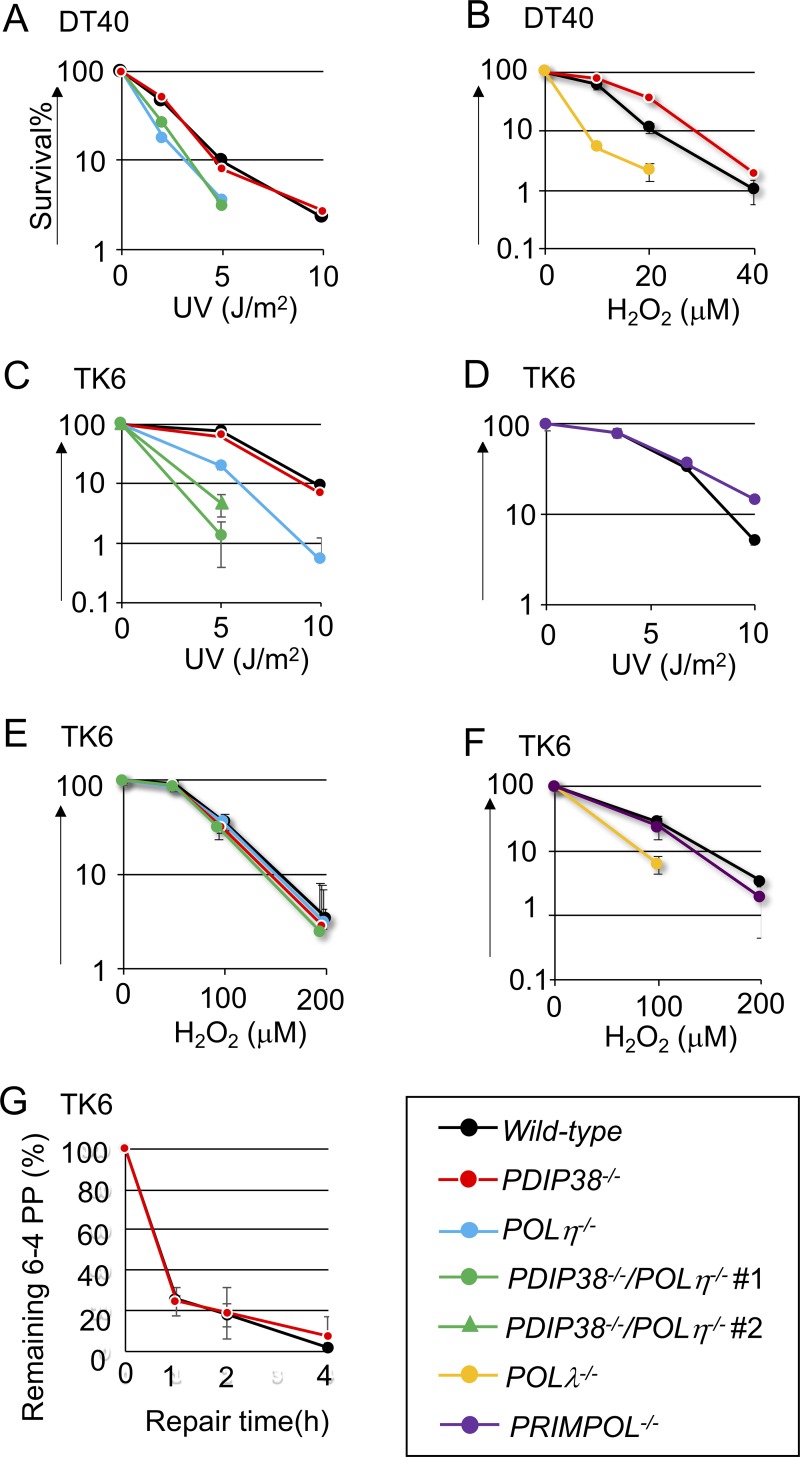
Cellular sensitivity to DNA-damaging agents. Chicken DT40 cells (A to B) and human TK6 cells (C to F) carrying the indicated genotypes were exposed to the indicated genotoxic agents. The dose of UV and H_2_O_2_ is displayed on the x-axis on a linear scale, while the percentage of colony survival is displayed on the y-axis on a logarithmic scale. Error bars show the standard deviation (SD) of mean for three independent assays. (G) Elimination of UV lesion (6–4 photoproducts) from genomic DNA. TK6 cells carrying the indicated genotypes were exposed to UV at time zero, and genomic DNA was isolated at the indicated time after UV irradiation and fixed on a microtiter plate. The relative amount of 6–4 photoproducts was determined with antibody against 6–4 photoproducts.

To explore a role of PDIP38 in TLS, we measured cellular sensitivity to UV and H_2_O_2_. *PDIP38*^*-/-*^ DT40 cells were tolerant to these damaging agents (Fig 1A and 1B and S7A Fig). This phenotype is in contrast with increased sensitivities of *POLη*^*-/-*^ and *PRIMPOL*^*-/-*^ cells to UV [34] (S8A Fig) and with increased sensitivity of *POLλ*^*-/-*^ cells to H_2_O_2_ [29]. *PDIP38*^*-/-*^ cells showed no significant sensitivity to cisplatin or methyl methanesulfonate (MMS) (S8B and S8C Fig). Thus, PDIP38 does not significantly affect the contribution of Polλ, Polη, and PrimPol to DDT in DT40 cells.

We next measured the sensitivities of *PDIP38*^*-/-*^ and *PDIP38*^*-/-*^*/POLη*^*-/-*^ TK6 cells to UV (Fig 1C and 1D and S7B Fig) and H_2_O_2_ (Fig 1E and 1F). *POLη*^*-/-*^ cells, but not *PDIP38*^*-/-*^ cells, were sensitive to UV, while *PDIP38*^*-/-*^*/POLη*^*-/-*^ cells were considerably more UV sensitive than *POLη*^*-/-*^ cells (Fig 1C). Thus, PDIP38 significantly contributes to UV tolerance independently of Polη in TK6 cells. We monitored the kinetics of nucleotide excision repair after UV irradiation by measuring the amount of 6–4 photoproducts, UV lesion, on the chromosomal DNA following UV-irradiation [48]. *PDIP38*^*-/-*^ cells showed normal kinetics of nucleotide excision repair (Fig 1G). Thus, the higher UV sensitivity of *PDIP38*^*-/-*^*/POLη*^*-/-*^ cells than that of *POLη*^*-/-*^ cells may be attributable to a defect in DDT.

*POLλ*^*-/-*^ TK6 cells, but not *PDIP38*^*-/-*^ or *PDIP38*^*-/-*^*/POLη*^*-/-*^ cells, were sensitive to H_2_O_2_ (Fig 1E and 1F). Thus, human PDIP38 does not enhance the functionality of Polλ in response to H_2_O_2_. In summary, PDIP38 significantly contributes to DDT through neither Polλ, Polη, nor PrimPol.

### *PDIP38*^*-/-*^ and *POLη*^*-/-*^ cells show increased sensitivity to replication stress

Aphidicolin, an inhibitor against replicative DNA polymerases, causes replication stress and induces mitotic chromosomal breaks at common fragile sites (CFSs) without actually generating double strand breaks (DSBs) [33, 49–51]. The paucity of replication origins increases the risk of incomplete replication upon replication stress. Polκ, Polη, and Polζ prevent the expression of CFSs [49, 52–54], suggesting the role played by these TLS polymerases in completion of DNA replication at CFSs.

**Fig 2:**
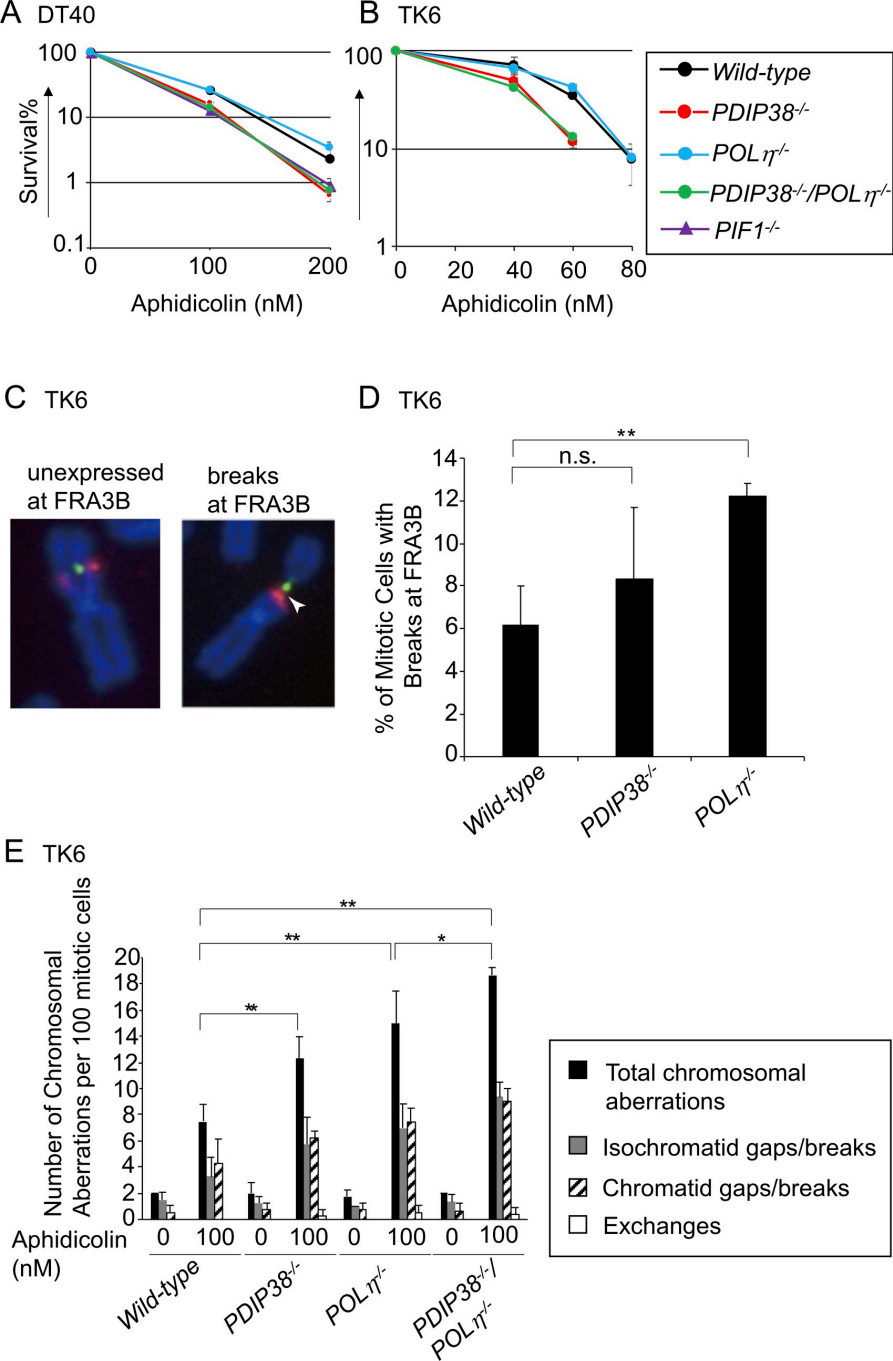
Contribution of PDIP38 to the prevention of chromosomal breakage after exposure to aphidicolin. Chicken DT40 cells (A) and human TK6 cells (B) with the indicated genotype were exposed to aphidicolin. The data are shown as in Fig 1. (C) Localization of the FRA3B locus was analyzed by using FISH (Red: RP11-495E23, Green: RP11-170K19). Arrowhead indicates a chromosomal breakage at FRA3B. (D) Quantification of mitotic chromosome breaks at FRA3B in *wild-type*, *POLη*^*-/-*^, and *PDIP38*^*-/-*^ TK6 cells (three experiments: >100 metaphases in each experiment), and SD are indicated by error bars. **: significant difference (by Student’s *t*-test) compared with *wild-type* cells (*P*<0.01). (E) The numbers of the indicated chromosomal aberrations per 100 mitotic cells carrying the indicated genotypes before (0 nM) and 48 h after treatment with 100 nM aphidicolin. Chromatid gaps/breaks indicate that these aberrations were seen in one of two sister chromatids, while isochromatid ones were seen at the same sites of both sisters. Error bars show the SD of mean for greater than or equal to three independent experiments. Statistical significance (by Student’s *t*-test) is as follow: ** *P*<0.01, * *P*<0.05.

We analyzed the role played by PDIP38 in cellular response to replication stress by measuring cellular sensitivity to aphidicolin. DT40 cells deficient in PIF1, a 5’-3’ DNA helicase required for efficient DNA replication at CFSs, are sensitive to aphidicolin as previously reported [33] (Fig 2A). The loss of PDIP38 caused a significant increase in the sensitivity to aphidicolin in both DT40 and TK6 cells. The disruption of Polη did not enhance the aphidicolin sensitivity in *wild-type* or *PDIP38*^*-/-*^ cells (Fig 2A and 2B). Nonetheless, since loss of Polη causes an increase in the number of aphidicolin-induced gaps/breaks (hereafter called chromosome breaks) at CFS sites in mitotic chromosome spreads [49], we measured induced chromosome breaks in *PDIP38*^*-/-*^ and *PDIP38*^*-/-*^*/POLη*^*-/-*^ TK6 cells. Chromosome breaks localized at the FRA3B locus, a typical CFS [54, 55], were detected in 8% of mitotic *PDIP38*^*-/-*^ cells (Fig 2C and 2D). The total numbers of induced chromosome breaks, which were calculated by subtracting the number of spontaneous breaks from the number of breaks seen in aphidicolin-treated cells, were 11 per 100 *PDIP38*^*-/-*^ mitotic cells (Fig 2E), indicating that a majority of the induced breaks occurred at FRA3B, which observation is consistent with a previous result [55]. Aphidicolin induced similar numbers of chromosomal breaks in *POLη*^*-/-*^, *PDIP38*^*-/-*^, and *PDIP38*^*-/-*^/*POLη*^*-/-*^ TK6 cells (Fig 2E), suggesting collaboration between Polη and PDIP38 in cellular response to replication stress. In conclusion, PDIP38 contributes to cellular response to replication stress and prevents expression of CFSs.

### *PDIP38*^*-/-*^ DT40 and TK6 cells show significant increases in the frequency of sister chromatid exchange

**Fig 3:**
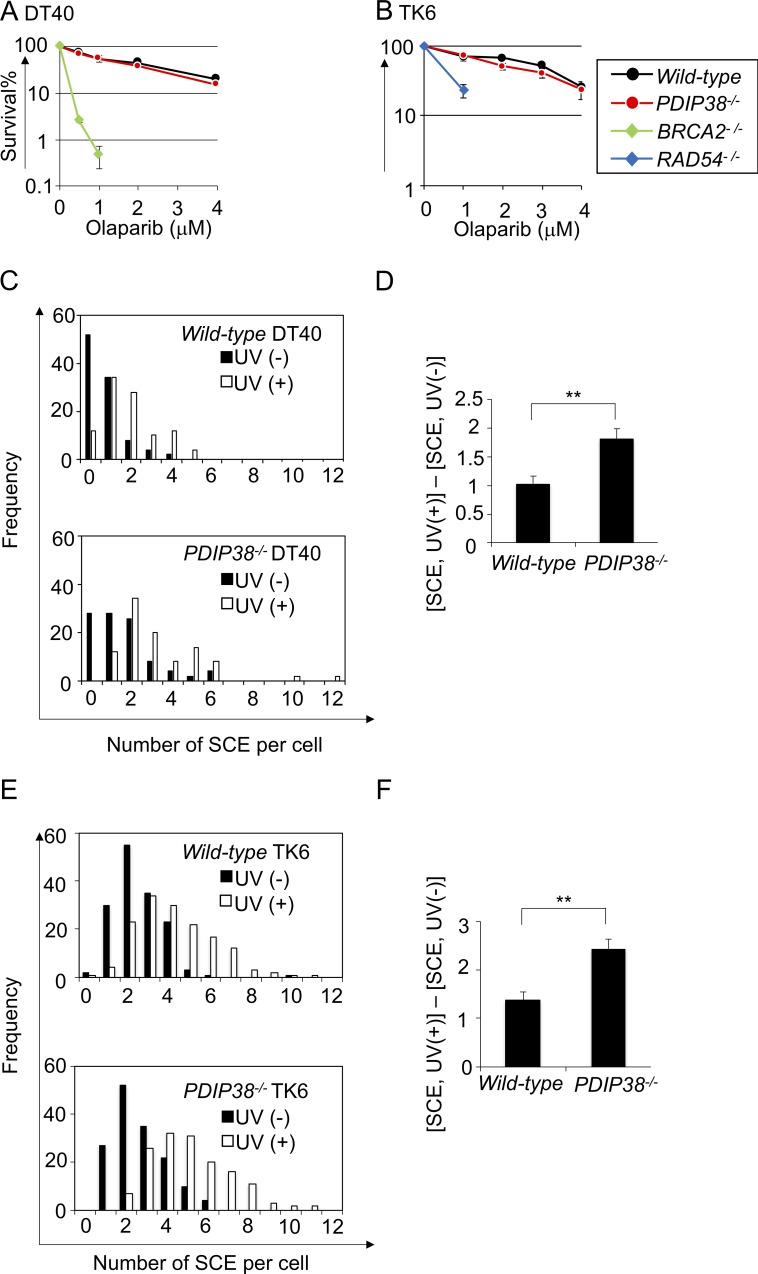
The loss of PDIP38 increases the frequency of SCE without affecting the capability of HR to perform DSB repair. Chicken DT40 cells (A) and human TK6 cells (B) with the indicated genotype were exposed to olaparib to evaluate the capability of HR to perform DSB repair. The data are shown as in Fig 1. (C) The number of SCE events of *wild-type* and *PDIP38*^*-/-*^ DT40 cells is indicated. Closed and open bars indicate the distribution of SCE/cell without UV treatment and with 0.25 J/m^2^ UV treatments, respectively. 50 cells were analyzed in each preparation. (D) The number of UV-induced SCE events (Y-axis) was calculated by subtracting spontaneous SCE events from SCE events following 0.25 J/m^2^ UV irradiation. Error bars show the SD in subtracted values obtained from at least three independent experiments. Statistical significance (by Student’s *t*-test) is as follow: ** *P*<0.01. (E and F) SCE was analyzed for human TK6 cells as in (C) and (D). TK6 cells were exposed to 5 J/m^2^ UV irradiation.

To evaluate the capability of *PDIP38*^*-/-*^ cells to carry out HR-dependent double-strand break (DSB) repair, we measured the sensitivity of *PDIP38*^*-/-*^ DT40 and TK6 cells to olaparib, a poly[ADP-ribose]polymerase inhibitor. Olaparib induces one-end breaks during DNA replication, which are repaired by HR [56]. As expected, cells deficient in HR factors, BRCA2 and Rad54, exhibit hypersensitivity to olaparib [57–59] (Fig 3A and 3B). In contrast, cellular sensitivity to olaparib was very similar between *PDIP38*^*-/-*^ and *wild-type* cells, in both DT40 and TK6 cell lines (Fig 3A and 3B), indicating that PDIP38 does not play an important role in HR.

We measured the number of SCE during the cell cycle, as well as after UV-irradiation (Fig 3C and 3E and S9 Fig). We then exposed cells to UV, and calculated the number of UV-induced SCE, i.e., the number of SCE in UV-irradiated cells subtracted by the number of spontaneously arising SCE. The number of the UV-induced SCE was more than 50% higher in *PDIP38*^*-/-*^ cells compared with *wild-type* cells in both the DT40 and TK6 cell lines (Fig 3D and 3F). In summary, PDIP38 may reduce the relative usage of TS in DDT both during unperturbed cell cycle and after UV irradiation.

### Loss of PDIP38 causes a shift of Ig V diversification from TLS to TS in the chicken DT40 B cell line

We next measured the usage of TLS-mediated nontemplated mutagenesis (Ig V hypermutation) and TS-dependent templated mutagenesis (Ig gene conversion) in DT40 cells (S1 Fig) in two conditions, a physiological expression level of AID and over-expressed AID [44]. In the former condition, Ig gene conversion dominates over Ig V hypermutation in the Ig V diversification. In the latter condition, on the other hand, an excess amount of AID-induced DNA lesions are processed by Ig V hypermutation in addition to Ig gene conversion.

**Fig 4:**
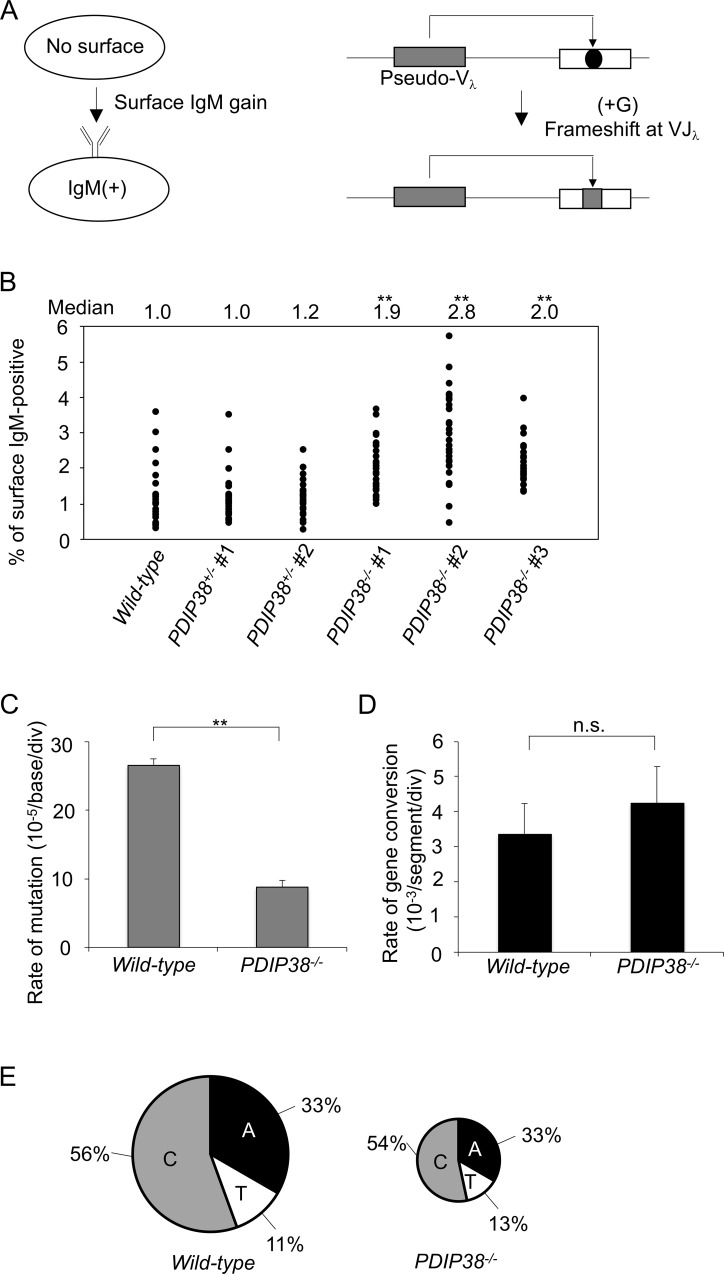
The loss of PDIP38 increases the frequency of TS mediated Ig gene conversion and reduces TLS mediated Ig V hypermutation. (A) The rate of Ig gene conversion can be estimated by measuring the rate of surface IgM gain during clonal expansion. Surface IgM negative cells contain a frameshift mutation (black dot) in the functional VJ_λ_ segment. The mutation is often repaired by upstream pseudo-V_λ_ segment-templated conversion events leading to surface IgM gain. (B) Fluctuation analysis of surface IgM gain in the six indicated genotypes. Median values of 30 subclones of each genotype are shown on top. Statistical significance of *P*<0.01 between *wild-type* and the three *PDIP38*^*-/-*^ clones (by Student’s *t*-test) is shown by **. The rates of TLS-mediated point mutations (PM) (C) and gene conversion (GC) (D) are indicated with standard error. Statistical significance (by Student’s *t*-test) of *P*<0.01 is shown by **. (E) Frequency of mutagenic base insertion of C, T, or A opposite C on either strand, corresponding to mutation from C to G, A and T, respectively. The size of the pie charts reflects the frequency of overall TLS-mediated point mutations within the examined nucleotide sequences, while the segments reflect the relative use of C, T, or A in bypass.

We firstly measured the rate of Ig gene conversion using the former condition. We generated *PDIP38*^*-/-*^ cells from the Cl18 DT40 variant clone, where a frameshift mutation at the V_λ_ segment inhibits the surface expression of IgM [23, 44]. Ig gene conversion events often repair the frameshift mutation, leading the gain of surface IgM expression (Fig 4A). The frequency of Ig gene conversion can be estimated from the fluctuation analysis of the surface IgM expression during clonal expansion of DT40 subclones. We made 30 subclones from each genotype, cultured them for three weeks, and measured the percentage of surface IgM positive cells in individual subclones. The median percentages of surface IgM positive cells were a few times higher in *PDIP38*^*-/-*^ cells when compared with *PDIP38*^*+/-*^ and *wild-type* cells (Fig 4B). Collectively, the analyses of SCE and Ig gene conversion indicate that PDIP38 changes the relative usage of DDT from TS to TLS. 　

We next over-expressed AID, isolated five over-expressed subclones in each genotype, and cultured them for two weeks to examine Ig V diversification during this clonal expansion period. We determined the Ig V_λ_ nucleotide sequences from the individual five subclones from *wild-type* and *PDIP38*^*-/-*^ DT40 cells. *PDIP38*^*-/-*^ cells showed a three-times decrease in the rate of TLS-mediated Ig V hypermutation when compared with *wild-type* cells even in the presence of excess numbers of DNA lesions (Fig 4C). We did not detect a further increase in the rate of Ig gene conversion in *PDIP38*^*-/-*^ cells in comparison to *wild-type* cells (Fig 4D), because AID-overexpression generates excessive numbers of DNA lesions, which fully stimulated TS irrespective of the status of PDIP38 expression. We conclude that PDIP38 enhances the usage of the DDT pathways from TS to TLS.

### Loss of PDIP38 causes a shift from TLS to TS in DDT to UV damage in TK6 cells

**Fig 5:**
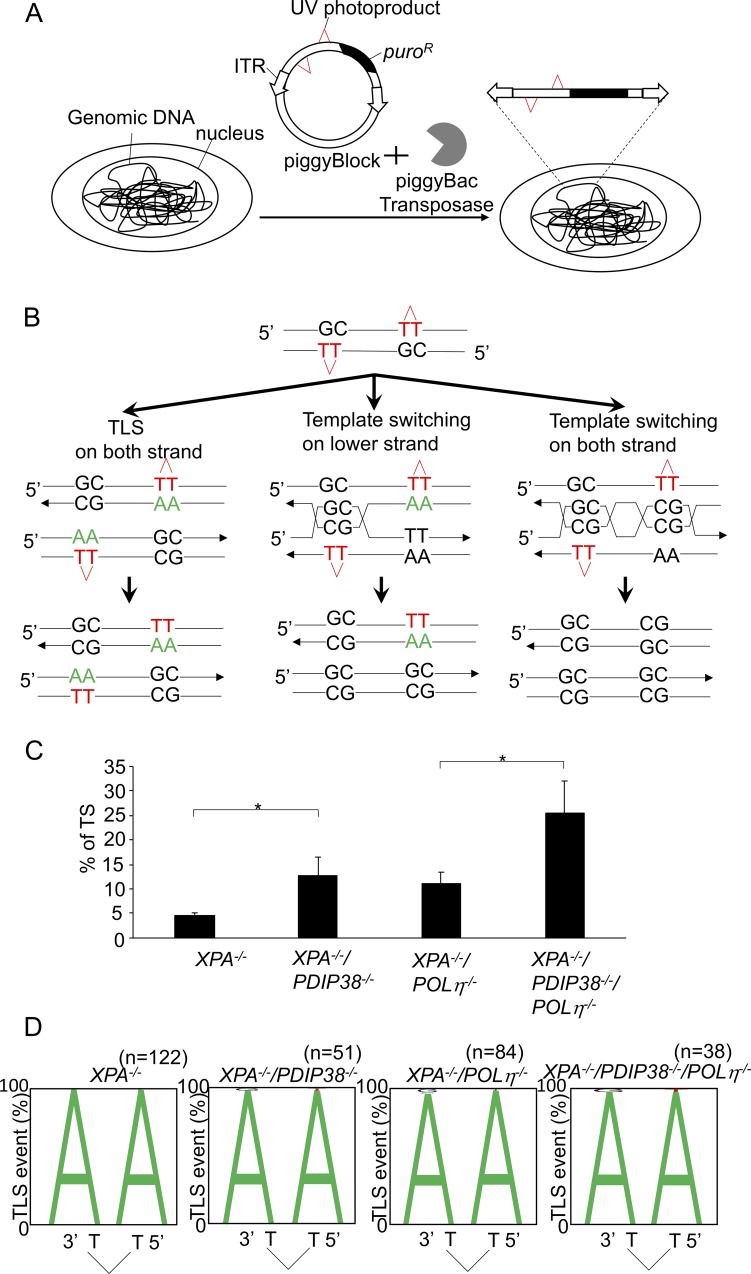
Loss of PDIP38 causes a shift from TLS to TS in bypassing the CPD UV damage. (A) Two CPDs placed opposite GpC mismatch were randomly integrated into the genome using the piggyBlock vector carrying *transposon*-specific inverted terminal repeat sequences (*ITRs*). (B) A schematic of the staggered arrangement of T–T(CPD) photoproducts with the dinucleotide GC placed opposite each lesion and 28 bp between the two lesions. Possible outcomes of DNA replication over the area as well as the pattern of nucleotide incorporation opposite the CPD site are shown. TLS may occur on either the top or the bottom strand. Alternatively, the nascent strand of the sister chromatid may be used as an alternative template, leading to TS. (C) The percentage of template switching (TS) events in the indicated genotypes. Error bars show the SD of mean for at least three independent experiments. Statistical significance (by Student’s *t*-test) is as follow: * *P*<0.05. (D) The pattern of TLS opposite the T–T(CPD) photoproduct. The percentage of nucleotides incorporated at the indicated positions is indicated by the size of the letter of the nucleotide in the column. The incorporation positions indicated are at the 3’-T and 5’-T of the lesion. The n number indicates the analyzed TLS events. The proportion of accurate TLS (incorporation of ApA opposite to CPD) was 100% in *XPA*^*-/-*^ cells. The following mutations were detected with frequencies shown in the parenthesis. G insertion (1/51) opposite the 3’-T and T insertion (1/51) opposite the 5’-T in *XPA*^*-/-*^*/PDIP38*^*-/-*^ cells. C insertion (1/84) and G insertion (2/84) opposite the 3’-T, and T insertion (1/84) opposite the 5’-T in *XPA*^*-/-*^*/POLη*^*-/-*^. G insertion (1/38) opposite the 3’-T and T insertion (1/38) opposite the 5’-T in *XPA*^*-/-*^*/PDIP38*^*-/-*^*/POLη*^*-/-*^.

We next investigated whether expression of PDIP38 changes the relative usage of TLS and TS in TK6 cells. To this end, we integrated UV lesions (CPD) into the genomic DNA using a transposon-based vector (piggyBlock assay system) carrying the puromycin resistance (*puro*^*R*^) gene [26](S2 Fig). To avoid elimination of integrated CPD by nucleotide excision repair, we disrupted the gene encoding *XPA*, a factor essential for nucleotide excision repair, in *wild-type* and *POLη*^*-/-*^ cells [60]. We then disrupted the *PDIP38* gene in *XPA*^*-/-*^ and *XPA*^*-/-*^*/POLη*^*-/-*^ cells. We transfected the piggyBlock vector carrying CPD into *XPA*^*-/-*^, *XPA*^*-/-*^*/PDIP38*^*-/-*^, *XPA*^*-/-*^*/POLη*^*-/-*^, and *XPA*^*-/-*^*/PDIP38*^*-/-*^*/POLη*^*-/-*^ cells (Fig 5A). We then selected cells with puromycin, PCR amplified nucleotide sequences over the CPD site in individual *puro*^*R*^ clones, and subjected individual amplified fragments to nucleotide sequence analysis.

To clearly distinguish TS from TLS, we modified the original piggyBlock assay system containing a single CPD by inserting another CPD into the vector. The resulting two CPDs of the piggyBlock vector were arranged in the staggered conformation following the design of a DDR reporter plasmid shown in a previous manuscript [60, 61]. The lesions were separated by 39 intervening base pairs and placed opposite a GpC mismatch (Fig 5B). Replicated copies result from either TLS or error-free TS on the top and bottom strands. TS is detectable by identifying GpC at the site of CPD, while TLS past CPD may insert ApA (accurate TLS) or other nucleotides (inaccurate TLS) (Fig 5B).

We determined the relative usage of TLS and TS to bypass the CPD damage site. The proportion of (GC) sequences was 5% for *XPA*^*-/-*^, 13% for *XPA*^*-/-*^*/PDIP38*^*-/-*^, 11% for *XPA*^*-/-*^*/POLη*^*-/-*^, and 26% for *XPA*^*-/-*^*/PDIP38*^*-/-*^*/POLη*^*-/-*^. Thus, the loss of PDIP38 causes a significant increase in the relative usage of TS, irrespective of the expression of Polη (Fig 5C). All genotypes had replicated almost exclusively by an error-free mechanism and generated only a few mutant sequences, even in the absence of Polη (Fig 5D). In summary, the analyses of SCE, Ig gene conversion, and bypass at the UV damage (CPD) consistently indicates that the loss of PDIP38 increases the usage of TS.

## Discussion

Here we provide genetic evidence that PDIP38 controls DDT by suppressing TS, as well as promoting TLS. We analyzed the Ig V diversification of chicken DT40 B cell line as this type of phenotypic analysis provides a unique advantage of accurately counting individual TLS and TS events past the abasic site (S1 Fig). *PDIP38*^*-/*-^ cells showed a shift in Ig V diversification from TLS (non-templated Ig V hypermutation) to TS (Ig gene conversion) (Fig 4). Human TK6 cells also showed a shift in bypass past the CPD UV lesion (S2 Fig) from TLS to TS (Fig 5). The loss of PDIP38 caused approximately two times increase in the number of UV-induced SCE (Fig 3). These data consistently indicate that PDIP38 increases the usage of TLS, while decreasing the usage of TS.

An important question is whether or not PDIP38 activates the TLS pathway? Depletion of PDIP38 sensitizes MRC5V1 cells to UV in the presence of Polη [15], indicating that PDIP38 can activate TLS in some cell lines. However, the following evidence argues against the activation of TLS by PDIP38, at least in DT40 and TK6 cell lines. First, although TLS copes with excessive amounts of environmental DNA damage, the loss of PDIP38 did not cause significant increases in cellular sensitivity to UV, H_2_O_2_, cisplatin, or MMS (Fig 1 and S8 Fig). The tolerance of *PDIP38*^*-/*-^ cells to these agents is in marked contrast with the phenotype of *RAD18*^*-/-*^ cells, which cannot efficiently activate TLS showing high sensitivities to a variety of DNA damaging agents [10]. Second, the tolerance of *PDIP38*^*-/*-^ cells is also in contrast with increased sensitivity of TLS polymerase mutants, *POLη*^*-/-*^, *POLλ*^*-/-*^, and *PRIMPOL*^*-/-*^ cells, to H_2_O_2_ and UV (Fig 1 and S8 Fig). Third, the loss of PDIP38 did not affect the mutation spectrum of TLS-mediated Ig V hypermutation (Fig 4E), which is in contrast with the data that loss of Polη, Polν, Polθ, Polζ, and Rev1 significantly changes the mutation spectrum [44, 60, 61]. One possible scenario is that PDIP38 might suppress TS and consequently increase the usage of TLS. TLS may bypass past a considerably larger number of DNA lesions in comparison with TS, which is carried out by a larger number of steps, including Rad51 polymerization, in comparison with TLS. This scenario thus explains the tolerance of *PDIP38*^*-/*-^ cells to various DNA lesions (Fig 1 and S8 Fig) though the usage of TLS was decreased in the absence of PDIP38 (Fig 4C). PDIP38 might suppress TS by interfering with physical interactions between PCNA and TS factors. Defining the molecular mechanisms by which PDIP38 shifts DDT from TS to TLS is an important question to be studied in the future.

Polη promotes fragile site stability under replication stress [49]. Here we have shown that PDIP38 deficiency increased sensitivity to aphidicolin in both chicken DT40 and human TK6 cells (Fig 2A and 2B), which phenotype is in contrast with no increased sensitivity to alkylating agents (MMS), H_2_O_2_, cisplatin, or UV. More than 50% of the induced chromosome breaks were observed at the FRA3B locus, a typical CFS site seen in lymphoid cells [54, 55], in both *POLη*^*-/-*^ and *PDIP38*^*-/*-^ mutants (Fig 2D and 2E). *PDIP38*^*-/*-^ and *POLη*^*-/-*^ TK6 cells displayed a few times increases in the numbers of aphidicolin-induced chromosomal breaks in comparison with *wild-type* cells (Fig 2E). Considering that the number of identified CFS breaks is 10–20 sites in various lineages of cells [50], very frequent breakage at the FRA3B locus (Fig 2D) indicates that the vast majority of the induced chromosome breaks represent CFSs in *POLη*^*-/-*^ and *PDIP38*^*-/*-^ mutants. Aphidicolin induced comparable numbers of chromosomal breaks in *POLη*^*-/-*^, *PDIP38*^*-/-*^, and *PDIP38*^*-/-*^/*POLη*^*-/-*^ TK6 cells (Fig 2E), suggesting collaboration between Polη and PDIP38 in cellular response to replication stress. In summary, PDIP38 may play an important role in the completion of DNA replication under replication stress.

## Supporting information

S1 Fig AID-dependent cytosine to uracil conversion initiates gene conversion and hypermutation in a chicken Ig V segment. (A) Activation induced deaminase (AID) deaminates cytosine converting it into uracil. This uracil base is removed by base excision repair leading to the formation of the abasic (AP) site. (B) Replication blockage at this site is released by TLS past abasic sites and TS to upstream pseudo-V_λ_ segments. TLS causes hypermutation at the G/C pair, and TS causes Ig gene conversion.
(TIFF)

S2 Fig Outline of the piggyBlock transposon-based system to analyze TLS past CPD UV photoproducts and TS events. CPD placed opposite GpC mismatch in the piggyBlock plasmid carrying the puromycin resistance (*puro*^*R*^) gene. After transfection, we immediately did limiting dilution of the cells in 96-well cluster plates followed by the selection of clones carrying the piggyblock plasmid randomly integrated into the genome using puromycin. Bypass by accurate TLS inserts the correct complementary base (AA) on lower strand at the damaged template base. Alternatively, the nascent strand of the sister chromatid is used as an alternative undamaged template; one possible mechanism for such a template switching illustrated.
(TIFF)

S3 Fig Gene targeting of the *PDIP38* locus in DT40 cells. (A) Schematic representation of the *PDIP38* locus in DT40 cells and the structure of the gene-targeting constructs. The open and close solid boxes indicate the non-coding and coding regions of exons, respectively. ‘S’ indicates relevant *Sac*II site. (B) Southern blot analysis of the *Sac*II-digested genomic DNA from cells carrying the indicated genotypes, using the probe shown in (A). The position and sizes of hybridizing fragments of the *wild-type* and targeted loci are indicated. (C) *Wild-type* as well as *PDIP38*^*-/-*^ DT40 cells were subjected to RT-PCR using *β-actin*- or *PDIP38*-specific primers. (D) The average doubling time for the indicated genotypes. Error bars show the standard error in at least three independent experiments.
(TIFF)

S4 Fig Gene targeting of the *PDIP38* locus in TK6 cells. (A) Schematic representation of the *PDIP38* locus in TK6 cells and the structure of the gene-targeting constructs. The open and close solid boxes indicate the non-coding and coding regions of exons, respectively. ‘N’ indicates relevant *Nhe*I site. (B) Southern blot analysis of the *Nhe*I-digested genomic DNA from cells carrying the indicated genotypes, using the probe shown in (A). The positions and sizes of hybridizing fragments of the *wild-type* and targeted loci are indicated. (C) *Wild-type* as well as *PDIP38*^*-/-*^ TK6 cells were subjected to RT-PCR using *GAPDH*- or *PDIP38*-specific primers. (D) The average doubling time for the indicated genotypes. Error bars show the standard error in at least three independent experiments.
(TIFF)

S5 Fig Gene targeting of the *POLλ* locus in TK6 cells. (A) Schematic representation of the *POLλ* locus in TK6 cells and the structure of the gene-targeting constructs. The close solid boxes indicate the coding regions of exons. Arrows are primers used for RT-PCR. (B) *Wild-type* as well as *POLλ*^*-/-*^ TK6 cells were subjected to RT-PCR using *GAPDH*- or *POLλ*-specific primers.
(TIFF)

S6 Fig Gene targeting of the *PRIMPOL* locus in TK6 cells. (A) Schematic representation of the *PRIMPOL* locus in TK6 cells and the structure of the gene-targeting constructs. (B) *Wild-type* as well as *PRIMPOL*^*-/-*^ TK6 cells were subjected to RT-PCR using *β*-actin- or *PRIMPOL*-specific primers.
(TIFF)

S7 Fig No increased sensitivity of *PDIP38*^*-/-*^ DT40 and TK6 cells to UV. DT40 cells (A) and TK6 cells (B) carrying the indicated genotypes were exposed to UV. Data are shown as in Fig 1.
(TIFF)

S8 Fig No increased sensitivity of *PDIP38*^*-/-*^ DT40 cells to cisplatin or MMS. (A to C) Colony survival of the indicated genotypes in the presence of UV(A), cisplatin (B), and MMS (C). Data are shown as in Fig 1. The data (A) is from [34].
(TIFF)

S9 Fig Number of spontaneous SCE and SCE following UV irradiation in *PDIP38*^*-/-*^ DT40 and TK6 cells. (A)The mean number of SCE per cell of *wild-type* and *PDIP38*^*-/-*^ DT40 cells is indicated. Error bars show the SD at least three independent experiments. Statistical significance (by Student’s *t*-test) is as follows: * *P*<0.05, ** *P*<0.01. (B) SCE was analyzed for human TK6 cells as in (A).
(TIFF)
