# Supplementary figures and images for: PDIP38/PolDIP2 controls the DNA damage tolerance pathways by increasing the relative usage of translesion DNA synthesis over template switching
Source: PLoS One. 2019 Mar 6;14(3):e0213383. doi: 10.1371/journal.pone.0213383 (PMC6402704; doi:10.1371/journal.pone.0213383)

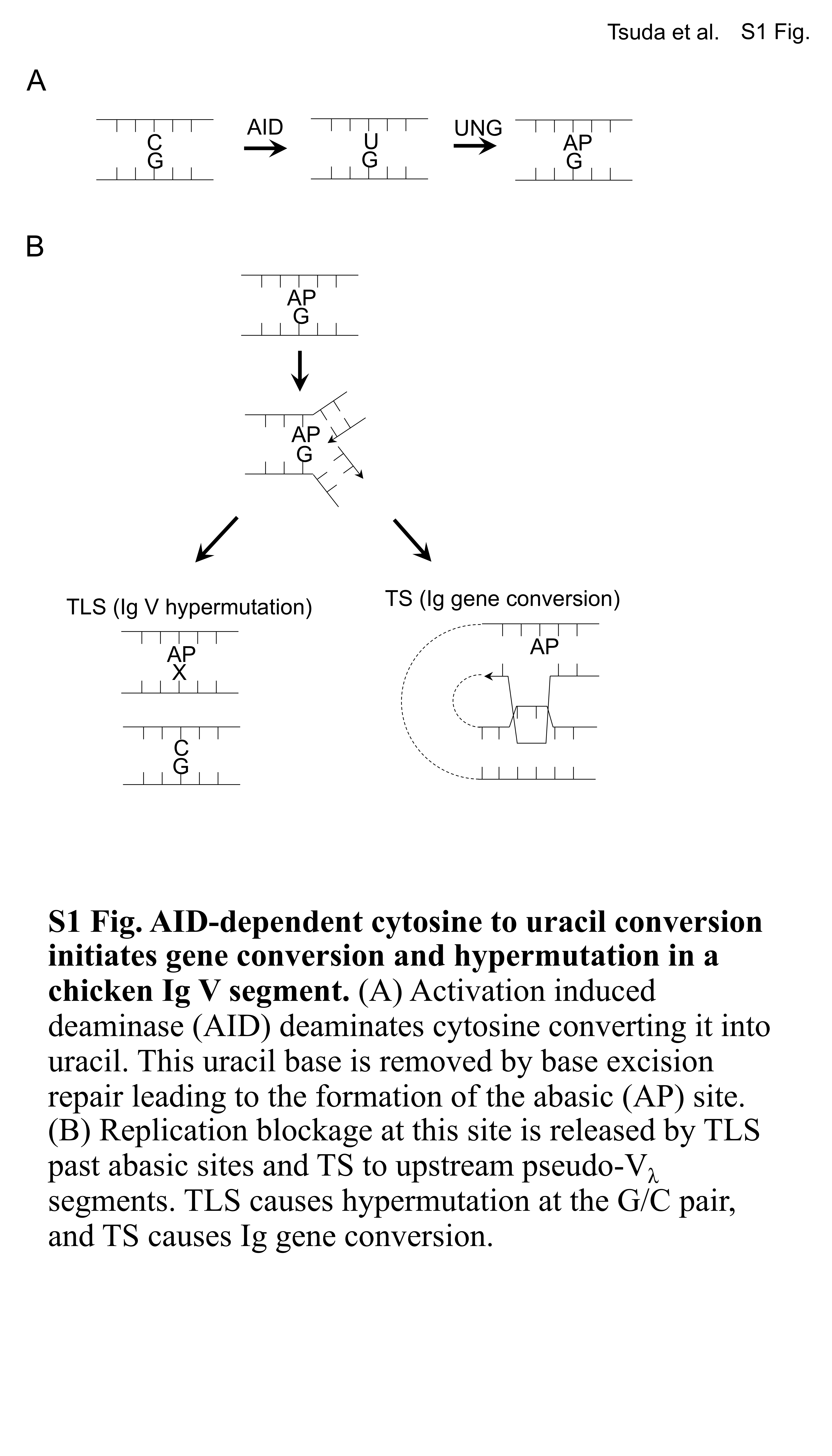

Supplement: S1 Fig — (A) Activation induced deaminase (AID) deaminates cytosine converting it into uracil. This uracil base is removed by base excision repair leading to the formation of the abasic (AP) site. (B) Replication blockage at this site is released by TLS past abasic sites and TS to upstream pseudo-Vλ segments. TLS causes hypermutation at the G/C pair, and TS causes Ig gene conversion. (TIFF) [file pone.0213383.s001.tiff]

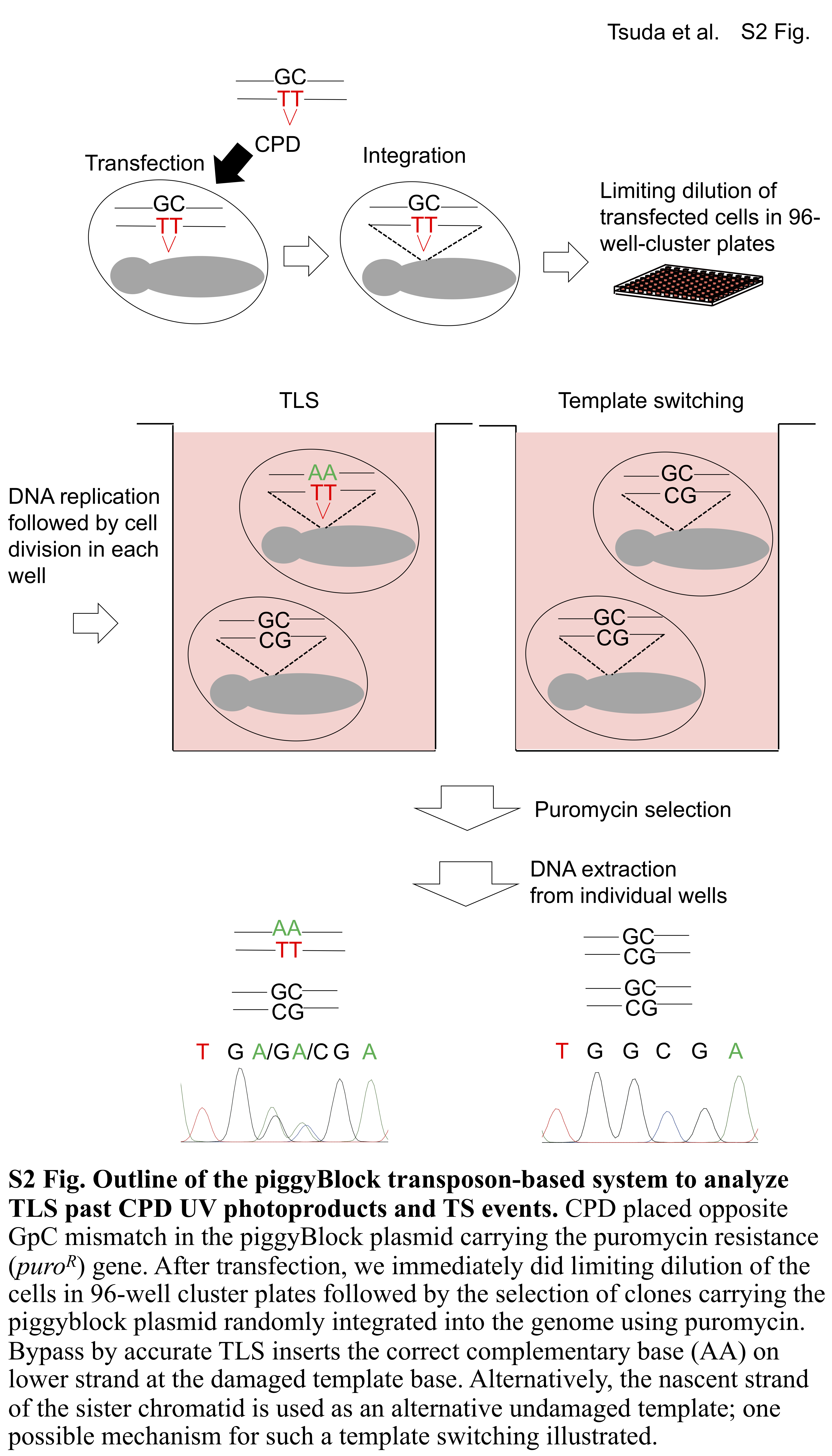

Supplement: S2 Fig — CPD placed opposite GpC mismatch in the piggyBlock plasmid carrying the puromycin resistance (puroR) gene. After transfection, we immediately did limiting dilution of the cells in 96-well cluster plates followed by the selection of clones carrying the piggyblock plasmid randomly integrated into the genome using puromycin. Bypass by accurate TLS inserts the correct complementary base (AA) on lower strand at the damaged template base. Alternatively, the nascent strand of the sister chromatid is used as an alternative undamaged template; one possible mechanism for such a template switching illustrated. (TIFF) [file pone.0213383.s002.tiff]

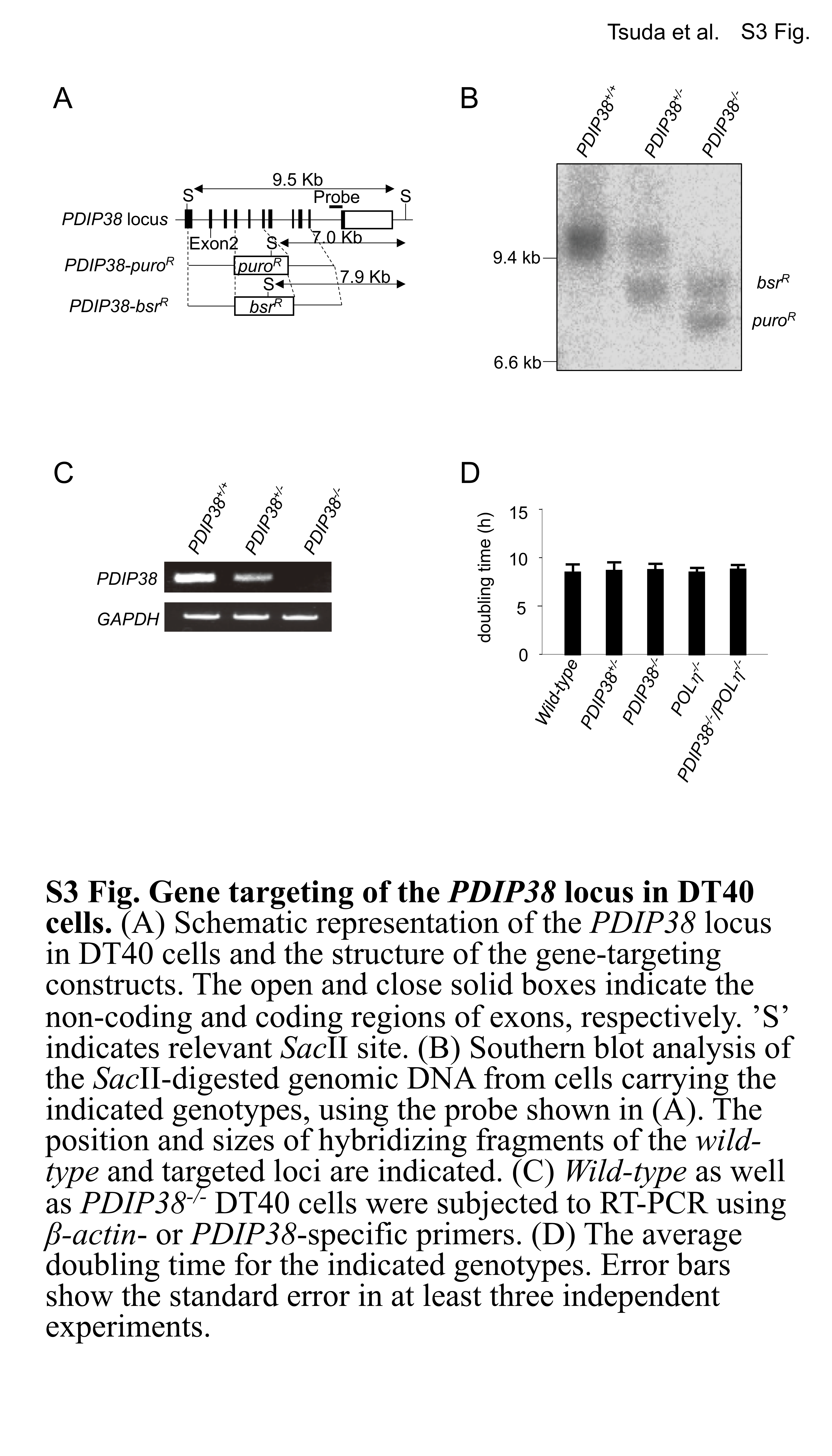

Supplement: S3 Fig — (A) Schematic representation of the PDIP38 locus in DT40 cells and the structure of the gene-targeting constructs. The open and close solid boxes indicate the non-coding and coding regions of exons, respectively. ‘S’ indicates relevant SacII site. (B) Southern blot analysis of the SacII-digested genomic DNA from cells carrying the indicated genotypes, using the probe shown in (A). The position and sizes of hybridizing fragments of the wild-type and targeted loci are indicated. (C) Wild-type as well as PDIP38-/- DT40 cells were subjected to RT-PCR using β-actin- or PDIP38-specific primers. (D) The average doubling time for the indicated genotypes. Error bars show the standard error in at least three independent experiments. (TIFF) [file pone.0213383.s003.tiff]

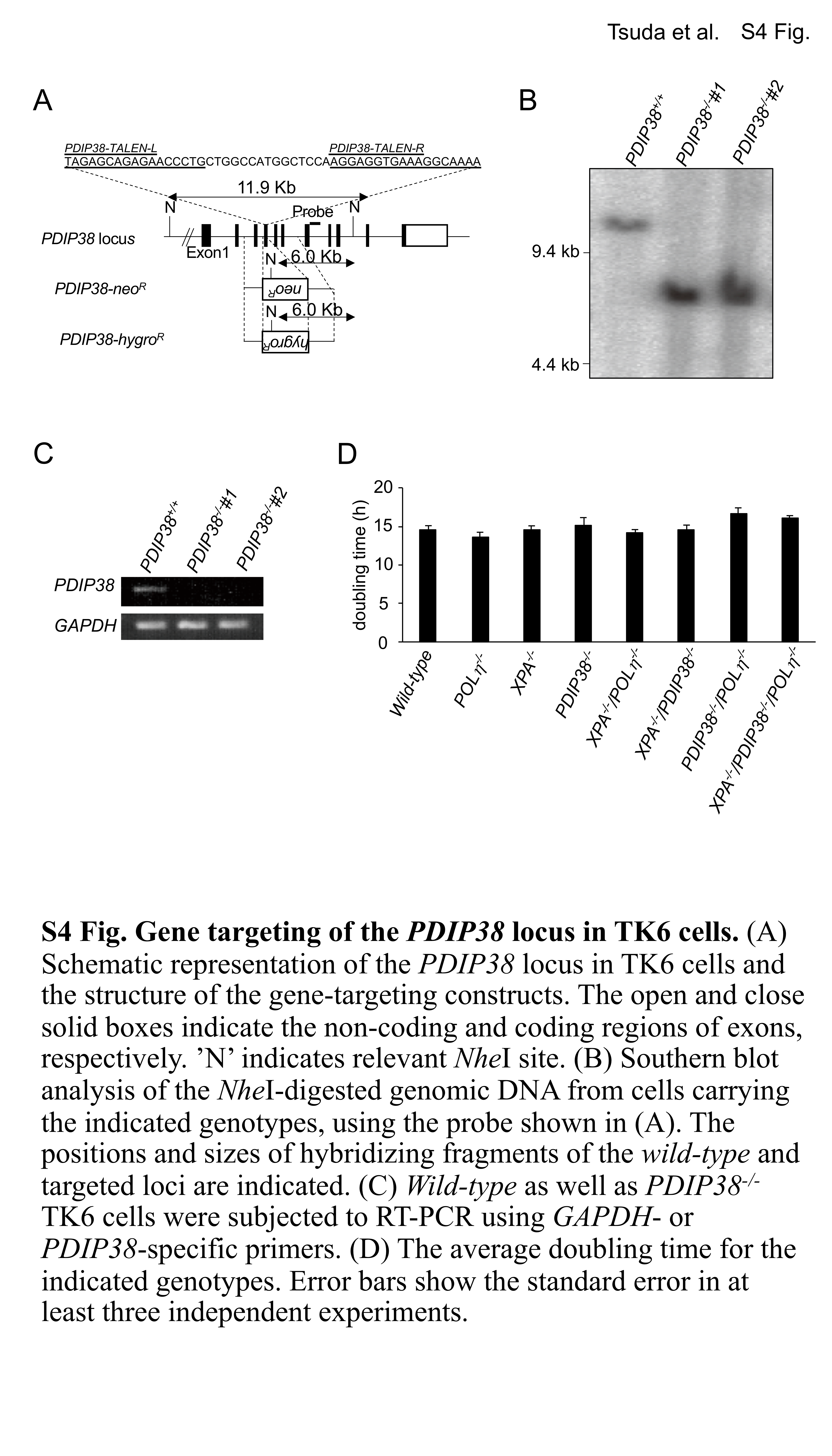

Supplement: S4 Fig — (A) Schematic representation of the PDIP38 locus in TK6 cells and the structure of the gene-targeting constructs. The open and close solid boxes indicate the non-coding and coding regions of exons, respectively. ‘N’ indicates relevant NheI site. (B) Southern blot analysis of the NheI-digested genomic DNA from cells carrying the indicated genotypes, using the probe shown in (A). The positions and sizes of hybridizing fragments of the wild-type and targeted loci are indicated. (C) Wild-type as well as PDIP38-/- TK6 cells were subjected to RT-PCR using GAPDH- or PDIP38-specific primers. (D) The average doubling time for the indicated genotypes. Error bars show the standard error in at least three independent experiments. (TIFF) [file pone.0213383.s004.tiff]

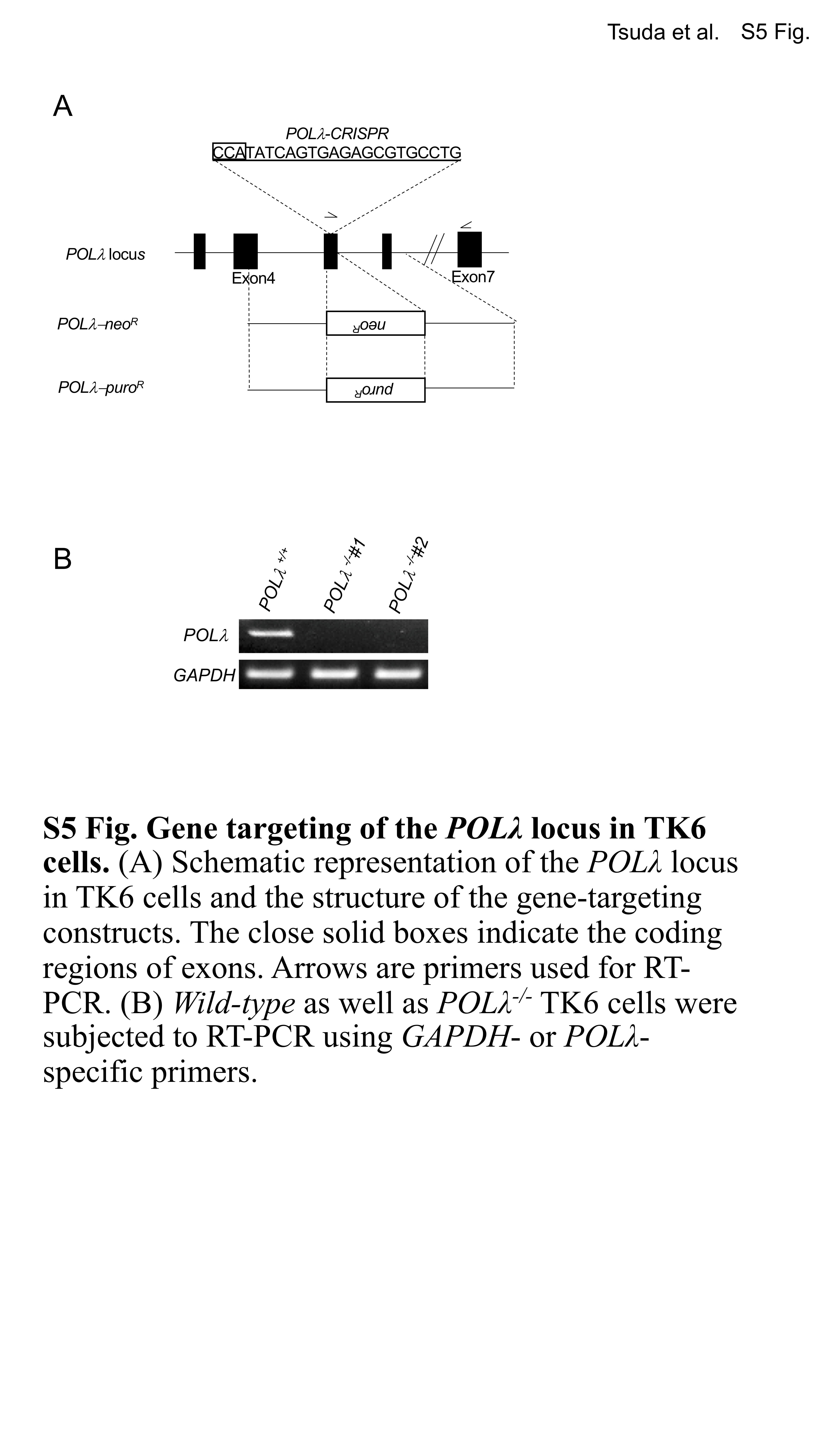

Supplement: S5 Fig — (A) Schematic representation of the POLλ locus in TK6 cells and the structure of the gene-targeting constructs. The close solid boxes indicate the coding regions of exons. Arrows are primers used for RT-PCR. (B) Wild-type as well as POLλ-/- TK6 cells were subjected to RT-PCR using GAPDH- or POLλ-specific primers. (TIFF) [file pone.0213383.s005.tiff]

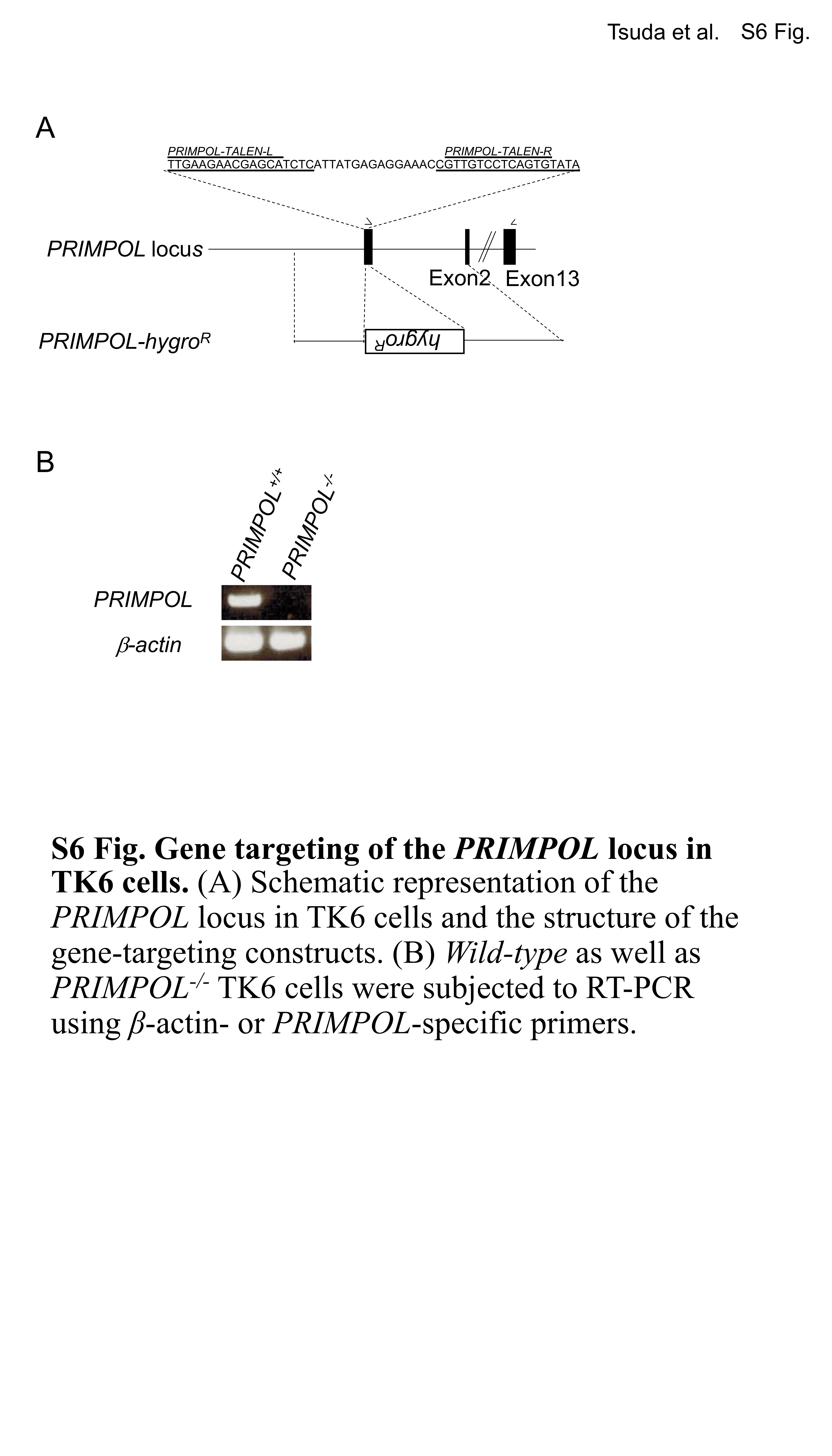

Supplement: S6 Fig — (A) Schematic representation of the PRIMPOL locus in TK6 cells and the structure of the gene-targeting constructs. (B) Wild-type as well as PRIMPOL-/- TK6 cells were subjected to RT-PCR using β-actin- or PRIMPOL-specific primers. (TIFF) [file pone.0213383.s006.tiff]

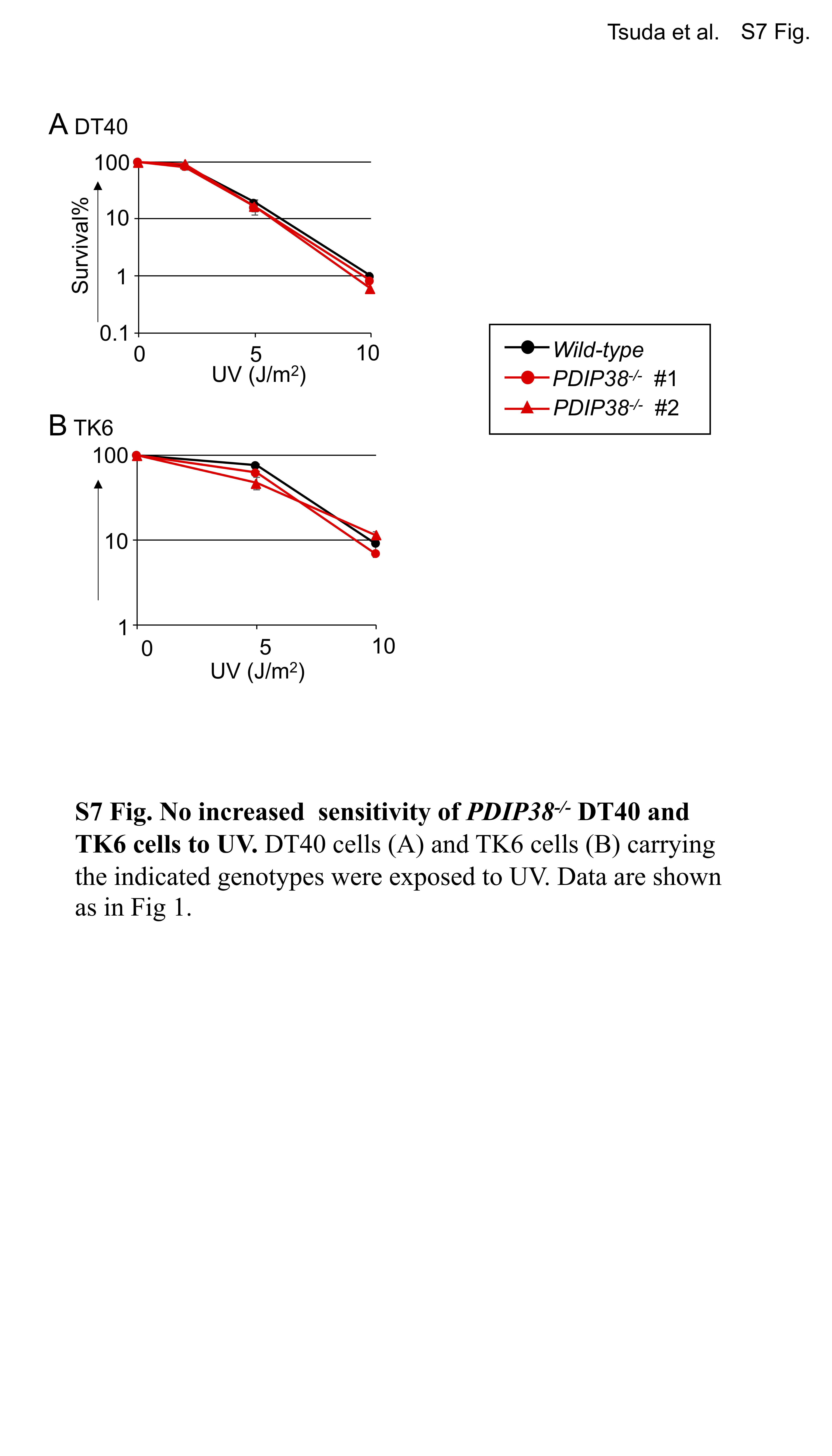

Supplement: S7 Fig — DT40 cells (A) and TK6 cells (B) carrying the indicated genotypes were exposed to UV. Data are shown as in Fig 1. (TIFF) [file pone.0213383.s007.tiff]

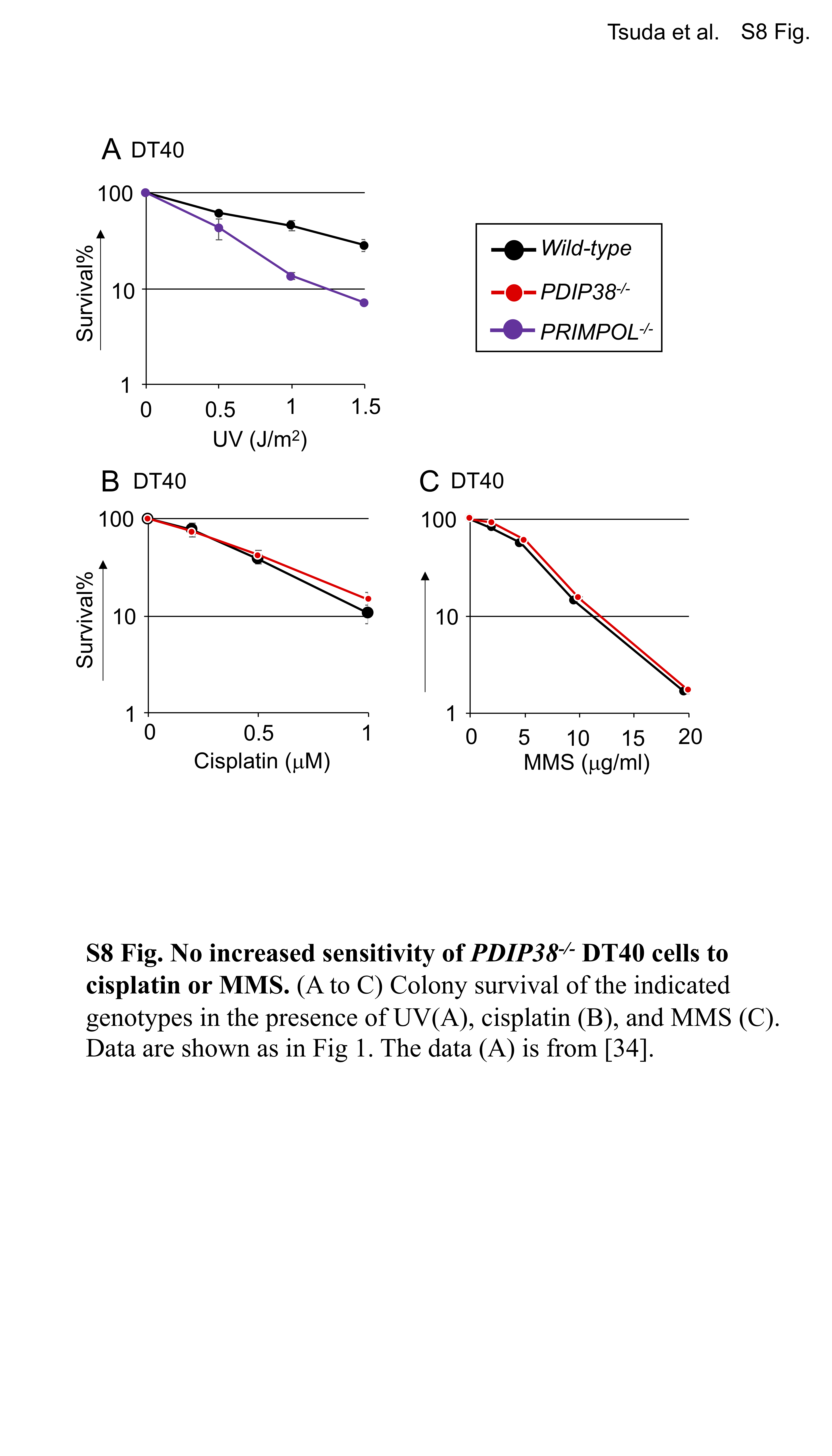

Supplement: S8 Fig — (A to C) Colony survival of the indicated genotypes in the presence of UV(A), cisplatin (B), and MMS (C). Data are shown as in Fig 1. The data (A) is from [34]. (TIFF) [file pone.0213383.s008.tiff]

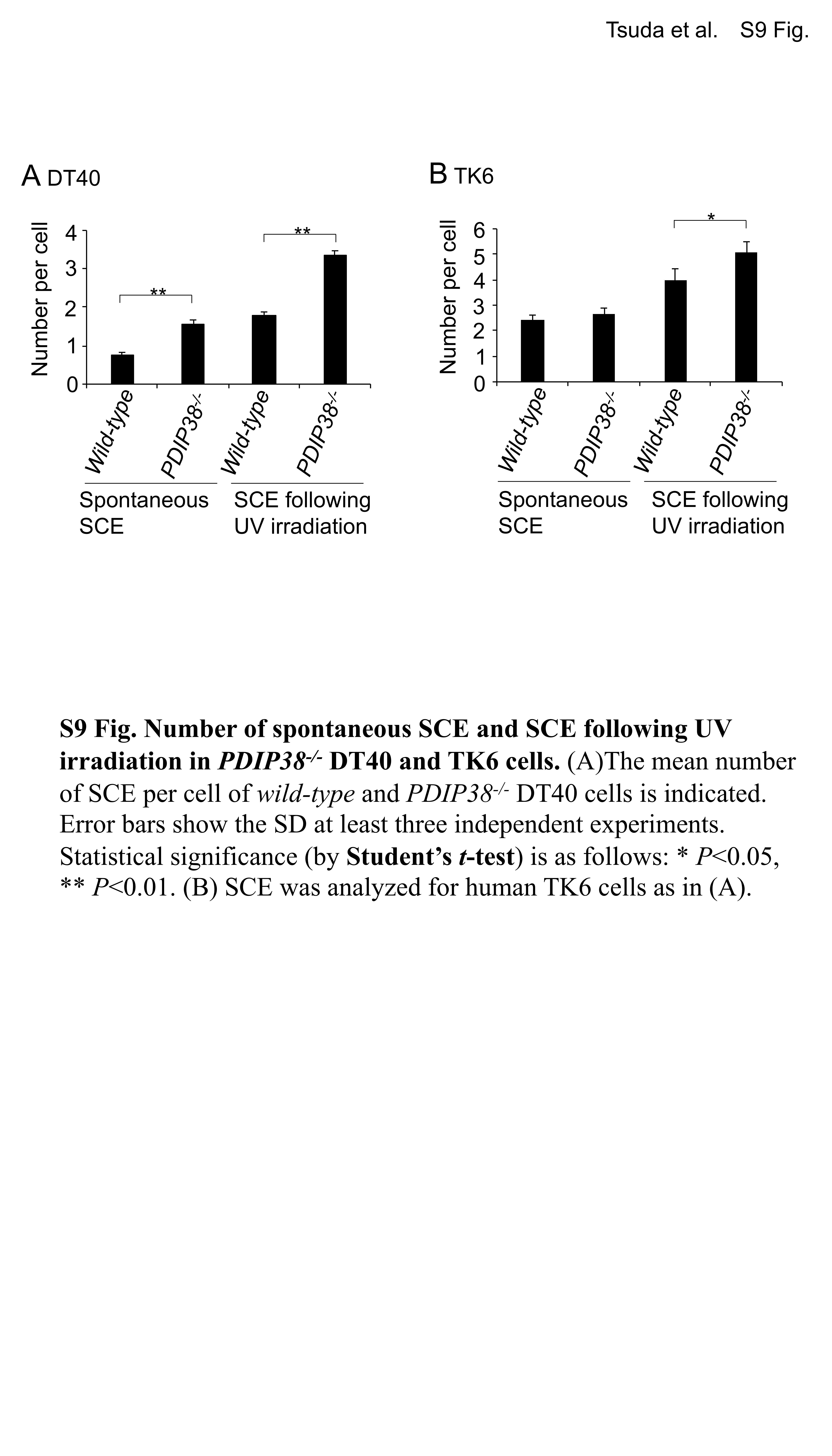

Supplement: S9 Fig — (A)The mean number of SCE per cell of wild-type and PDIP38-/- DT40 cells is indicated. Error bars show the SD at least three independent experiments. Statistical significance (by Student’s t-test) is as follows: * P<0.05, ** P<0.01. (B) SCE was analyzed for human TK6 cells as in (A). (TIFF) [file pone.0213383.s009.tiff]
